# Adaptations to high pressure of *Nautilia* sp. strain PV‐1, a piezophilic Campylobacterium (aka Epsilonproteobacterium) isolated from a deep‐sea hydrothermal vent

**DOI:** 10.1111/1462-2920.16256

**Published:** 2022-10-31

**Authors:** Francesco Smedile, Dionysis I. Foustoukos, Sushmita Patwardhan, Kelli Mullane, Ian Schlegel, Michael W. Adams, Gerrit J. Schut, Donato Giovannelli, Costantino Vetriani

**Affiliations:** ^1^ Department of Marine and Coastal Sciences Rutgers University New Brunswick New Jersey USA; ^2^ Institute of Polar Science (ISP‐CNR) Messina Italy; ^3^ Earth and Planets Laboratory Carnegie Institution of Washington Washington District of Columbia USA; ^4^ Marine Biology Research Division Scripps Institution of Oceanography La Jolla California USA; ^5^ Department of Biochemistry and Microbiology Rutgers University New Brunswick New Jersey USA; ^6^ Department of Biochemistry & Molecular Biology University of Georgia Athens Georgia USA; ^7^ Department of Biology University of Naples “Federico II” Naples Italy

## Abstract

Physiological and gene expression studies of deep‐sea bacteria under pressure conditions similar to those experienced in their natural habitat are critical for understanding growth kinetics and metabolic adaptations to in situ conditions. The Campylobacterium (aka Epsilonproteobacterium) *Nautilia* sp. strain PV‐1 was isolated from hydrothermal fluids released from an active deep‐sea hydrothermal vent at 9° N on the East Pacific Rise. Strain PV‐1 is a piezophilic, moderately thermophilic, chemolithoautotrophic anaerobe that conserves energy by coupling the oxidation of hydrogen to the reduction of nitrate or elemental sulfur. Using a high‐pressure–high temperature continuous culture system, we established that strain PV‐1 has the shortest generation time of all known piezophilic bacteria and we investigated its protein expression pattern in response to different hydrostatic pressure regimes. Proteogenomic analyses of strain PV‐1 grown at 20 and 5 MPa showed that pressure adaptation is not restricted to stress response or homeoviscous adaptation but extends to enzymes involved in central metabolic pathways. Protein synthesis, motility, transport, and energy metabolism are all affected by pressure, although to different extents. In strain PV‐1, low‐pressure conditions induce the synthesis of phage‐related proteins and an overexpression of enzymes involved in carbon fixation.

## INTRODUCTION

Since their discovery in 1977, deep‐sea hydrothermal vents have profoundly changed our view of life on Earth (Jannasch & Mottl, 1985; Jebbar et al., 2015; Paull, 1984). In particular, studies of the microbiology of deep‐sea hydrothermal vents highlighted the ability of microbes to thrive in extreme environmental conditions (Jebbar et al., 2015; Sievert & Vetriani, 2012). Deep‐sea hydrothermal vents are highly dynamic environments characterized by steep thermal and redox gradients, where temperature, oxygen concentration and redox potential can change dramatically over short space and time spans (Foustoukos et al., 2011; Jannasch & Mottl, 1985; Jebbar et al., 2015; Oger & Jebbar, 2010). In these environments, hydrothermal alteration of the oceanic crust results in the formation of anoxic vent fluids enriched in dissolved gasses (H_2_S, CH_4_, CO, CO_2_ and H_2_), and metals (Mn^2+^, Fe^2+^, Si^+^, Zn^2+^, etc.) (Charlou et al., 2002; Edmond et al., 1982; Charlou et al., 2002, 2010; Jannasch & Mottl, 1985; Johnson et al., 1986; Von Damm, 2000).

The mixing of hot, reduced hydrothermal fluids with cold, oxidized seawater provides a redox interphase where the continuous supply of electron donors and acceptors is harnessed by microorganisms to conserve energy and fix inorganic carbon (e.g. CO_2_, HCO_3_^−^; Jannasch & Mottl, 1985). Chemolithoautotrophic microorganisms at deep‐sea hydrothermal vents mediate primary production and are the foundation of the vent ecosystem (Sievert & Vetriani, 2012). In addition to steep thermal and redox gradients, deep‐sea vents are also characterized by high hydrostatic pressure (>10 MPa at >1000 m water depth), which impacts the structure and function of several cellular components, including membrane fluidity and the expression, structure and catalytic activity of enzymes (Oger & Jebbar, 2010). Hence, deep‐sea vent microorganisms are uniquely adapted to cope with the simultaneous effects of high pressure and high temperature (Amrani et al., 2014; Michoud & Jebbar, 2016). High‐pressure adapted organisms—piezophiles—exhibit a phylogenetic diversity that extends to all three domains of life (Jebbar et al., 2015).

Genomes obtained from pure cultures and single cells, as well as metagenomes from natural microbial communities, provide useful information to understand the metabolic potential of deep‐sea vent microorganisms (Giovannelli et al., 2017; Kaster & Sobol, 2020; Patwardhan et al., 2021). However, despite the progress in ‐omics techniques, little is known about the molecular mechanisms that confer physiological adaptations of microorganisms to high pressure (Jebbar et al., 2015). To this end, the study of pure cultures isolated in the laboratory is necessary (Takai et al., 2008). The information gleaned from studies of the effect of pressure on microbial physiology and metabolism is crucial to understand the role of primary producers that inhabit these unique environments (McNichol et al., 2018).

Studies of the impact of pressure on piezo‐sensitive microbes (organisms whose growth is negatively affected by increasing pressure) revealed the activation of different stress‐response mechanisms, including expression of cold and hot chaperones and mechanisms of homeoviscous adaptation to maintain the adequate fluidity of the cell membrane (Bartlett, 2002; Vezzi et al., 2005; Yang et al., 2007). For instance, early studies of the piezophilic bacterium, *Photobacterium profundum* SS9, showed the importance of outer membrane proteins in response to high‐pressure exposure and in signal transduction (Bartlett et al., 1996; Chi & Bartlett, 1993). Genomic and transcriptomic analyses of *P. profundum* SS9 further revealed that, at high pressure, the genes involved in fermentative processes and trimethylamine *N*‐oxide anaerobic respiration were upregulated—possibly in response to less efficient cytochrome oxidase activity—and that the metabolic pathways for the degradation of complex carbohydrates were induced at 28 MPa and suppressed at 0.1 MPa (Vezzi et al., 2005). More recent work on the thermopiezophilic bacterium, *Desulfovibrio hydrothermalis* (Amrani et al., 2014) and the obligate piezophilic and hyperthermophilic archaeon, *Pyrococcus yayanosii* (Michoud & Jebbar, 2016), showed that systems involved in translation, chemotaxis, energy metabolism, transport of solutes and amino acids, amino acid metabolism, stress‐response, and homeoviscous adaptation were influenced by pressure (Amrani et al., 2014; Jebbar et al., 2015; Michoud & Jebbar, 2016).

The effects of simultaneous osmotic and hydrostatic stress on microbes have not been extensively studied. However, there is some evidence linking them together: in *P. profundum* SS9, cellular inventory of multiple osmolytes is highest under high pressure and high salt concentrations, which seem to trigger the production of beta‐hydroxybutyrate (Martin et al., 2002). Further, *Halobacterium salinarum*, a halophilic piezosensitive archaeon, when grown at salinities sufficient to prompt its ‘salt in’ strategy was found to experience no significant reduction in viability following subsequent extended exposure to elevated pressure. In contrast, *Chromohalobacter salexigens*, a halophilic bacterium that employs the ‘salt out’ strategy, was found to be equally piezosensitive no matter the salinity (Kish et al., 2012).

Chemolithoautotrophic *Campylobacterota* (aka *Epsilonproteobacteria*) (Chun et al., 2018) have been identified as important members of hydrothermal vent microbial communities and play a critical role in primary productivity in marine geothermal habitats (Sievert & Vetriani, 2012). However, to date, studies of deep‐sea vent *Campylobacterota* have been carried out at ambient pressure. In this study, we report the isolation of the first piezophilic *Campylobacterium*, *Nautilia* sp. strain PV‐1, and its growth kinetics at high pressure. Further, we integrated genomic and proteomic approaches to investigate the expression profiles of strain PV‐1 during growth at ambient and elevated pressure. This work was facilitated by the recent development of techniques to continuously grow microorganisms under in situ pressure and temperature conditions (Foustoukos & Pérez‐Rodríguez, 2015; Houghton et al., 2019).

## EXPERIMENTAL PROCEDURES

### Enrichment, isolation and phylogenetic characterization

*Nautilia* sp. strain PV‐1 was isolated from hydrothermal fluids released from an *Alvinella pompejana*‐colonized active vent at 9° N on the East Pacific Rise (9°50.3981 N; 104°17.4942 W) during the R/V Atlantis AT26‐10 expedition (January 2014). The fluid temperature at the vent orifice was about 55°C. Vent fluids were collected using isobaric gas‐tight samplers (Seewald et al., 2002) during ROV *Jason* dive 761. The fluid samples were then transferred isobarically to a shipboard continuous culture system (Foustoukos & Pérez‐Rodríguez, 2015) and incubated at 25 MPa and 55°C (Foustoukos, 2015). Subsamples from these incubations were inoculated into stoppered Hungate tubes, each containing 10 ml of modified SME medium (Vetriani et al., 2004; Grosche et al., 2015) supplemented with 10% (w/v) nitrate under a H_2_/CO_2_ gas phase (80:20; 200 kPa) at atmospheric pressure. These primary enrichments were subjected to four consecutive series of dilutions to extinction. The culture obtained from the highest dilution of the fourth series was checked for purity by direct microscopic observation and by sequencing the 16 S ribosomal RNA (rRNA) gene. During the isolation procedure, direct counts of cells stained with acridine orange (0.1% w/v) were determined by visualization on an Olympus BX 60 microscope with an oil immersion objective (UPlanFl 100/1.3).

The 16 S rRNA gene of the resulting culture, which was named strain PV‐1 (Pressure Vessel‐1), was amplified by PCR and sequenced as described previously (Vetriani et al., 2004). The 16 S rRNA gene sequence of strain PV‐1 and close relatives were initially aligned using the SILVA alignment tool (Pruesse et al., 2007) and manually inserted in ARB (Ludwig et al., 2004). The sequences were then aligned using the SILVA databases for ARB (release 132 SSURef NR99). The neighbour‐joining algorithm and the Jukes–Cantor distance matrix of the ARB package were used to generate phylogenetic trees based on distance analysis. One thousand bootstrap re‐samplings were performed to estimate the robustness of the tree using the same distance model. This 16 S rRNA gene‐based phylogenetic analysis showed that the strain PV‐1 was an *Campylobacterium* of the family *Nautiliaceae*.

### Growth in the continuous culture system

Strain PV‐1 was maintained in the chemostat for a total of 66 days. To avoid excessive stress to the organisms, the experiment was separated in three different time periods: (i) 0–124 h: the organism was acclimated to the chemostat condition and growth rates at 0.4 MPa was measured; (ii) 142–1036 h: the pressure was increased up to 20 MPa; and (iii) 1462–1633 h: continuous culturing was performed at 0.5 MPa to investigate the effect of different dilution rates (flow rate/culture volume) on the growth rate of strain PV‐1, the experiment was conducted for 12‐h periods at each dilution rate, alternated by overnight incubations at the minimum flow rate of 0.2 ml min^−1^. During each 12‐h period, three replicate biomass samples were collected every 2 h. After each 12‐h period, the flow rate was increased at the new value, and the process was repeated. The fluctuations of cell concentrations shown in Figure 4 are mainly due to change in flow rate from day and night, while the value recorded at the different peaks is the average of three different samples obtained at the same flow rate. Specific growth rates (per hour) were determined by the change per unit of cell concentration as a function of time:

$$\mu =1/\mathrm{CX}dC/dT=\ln\left(2\right)/\mathrm{td}$$

where *C* is the cell density (number of cells per millilitre) and *td* is the doubling time in hours. The growth rate (*μ*) was calculated by the slope of the linear function between the natural logarithm values of cell densities and incubation time. The maximum growth rate of strain PV‐1 in the chemostat is described by the Monod equation (Foustoukos & Pérez‐Rodríguez, 2015 and reference therein) that correlate the dilution rate and substrate availability with microbial growth during continuous culture:

$$\mu =\mu_{\max}\left(S/K_{s}+S\right)$$

where *μ*_max_ is the maximum growth rate, *S* is the concentration of the growth‐limiting substrate measured at the outflow of the biochemostat, and *K*_*s*_ is the substrate concentration at which the growth rate equals *μ*_max_/2 (Foustoukos & Pérez‐Rodríguez, 2015).

### Analytical methods

Analysis of dissolved aqueous species and volatiles: Samples were collected with gas‐tight syringes. The concentrations of dissolved H_2_ and CO_2_ were determined by an SRI 8610C gas chromatography equipped with thermal conductivity detector/flame ionization detectors and a Carboxen‐1010 Plot/Silica‐Gel column. To measure the total CO_2(aq)_ concentrations (i.e. CO_2_ + HCO_3_^−^ + CO_3_^2−^), samples were introduced in gas‐tight syringes pre‐acidified with <50 μl of 2 M HCl. The detection limit for these volatiles is 5 μmol kg^−1^ and the analytical errors (2σ) are estimated to be less than 15%. Samples analysed for NO_3_^−^, NO_2_^−^ and NH_4_^+^ were passed through 0.2 μm pore‐size RC syringe filters and they were treated with 10 μl of 2 M HCl before being stored at −20° C. NO_3_^−^ and NO_2_^−^ concentration measurements were performed using ion chromatography (Metrohm, MIC‐3 Advanced IC, Metrosep A supp 7–250 column). NH_4_ = concentration measurements involved the alkaline hypochlorite/phenol nitroprusside method and the use of the Tecan Infinite M200 spectrophotometer (Pérez‐Rodríguez et al., 2017). The estimated uncertainty (2 σ) for the NO_3_^−^, NO_2_^−^ and NH_4_^+^ analysis is less than 2%. Dissolved NO_2_^−^ was absent in all the samples.

Stable N isotope analysis of dissolved NO_3_^−^ and NH_4_^+^: The δ^15^N composition of NO_3_^−^ and NH_4_^+^ was determined by following protocols discussed in the studies by Rodríguez et al. (2017). In brief, NH_4_^+^ dissolved in the aqueous samples was converted to NH_3(g)_ using a pH‐buffered Na2B4O7‐NaOH solution (pH = 12.7) and then diffused into the so‐called diffusion packets (Holmes et al., 1998; Pérez‐Rodríguez et al., 2017; Sigman et al., 1997). Diffusion packets are made with 1 cm diameter glass fibre grade GF/D filters, pre‐acidified with 25 μl of 2 M sulfuric acid (4 N) and sandwiched between 2.5 cm diameter 10 μm pore‐size Teflon membranes. Samples were incubated for 7 days at room temperature. After removing the diffusion packets, the GF/D discs were oven‐dried at 55°C. The same protocol was followed for the NO_3_^−^ after conversion to NH_4_^+^ using Devarda's alloy.

The stable N isotope composition of the NH_4_‐bearing GF/D discs was measured with a Thermo Scientific Delta VPlus mass spectrometer interfaced with a Carlo Erba (NA 2500) elemental analyser via a Conflo III interface. N_2_ references gas was introduced via the Conflo III (Pérez‐Rodríguez et al., 2017). Internal working gas standards were analysed at regular intervals during analysis to monitor the internal precision of the measured isotopic ratios and elemental compositions throughout the run. The reported uncertainties for the elemental and isotopic analyses correspond to 1 deviation between replicate analyses of distinct subsamples (*n* > 2). All data are reported in ™notation, in units of permil (‰) relative to N_2_ in air for ™^15^N = [(*R*_sample_/*R*_air_)‐1] *1000 ‰, where *R* = ^15^ N/^14^ N (Holmes et al., 1998; Pérez‐Rodríguez et al., 2017; Sigman et al., 1997).

### Genome sequencing and assembly

The genome of strain PV‐1 was sequenced using a combination of Illumina and MinION platforms. Cells were harvested after 24 h of growth and DNA was extracted immediately using the phenol–chloroform method described by Giovannelli et al. (2012). To minimize DNA shearing, mixing was done by inverting the tubes rather than vortexing. DNA concentration was quantified fluorometrically using the Invitrogen QuBit 2.0 Fluorometer and the Qubit dsDNA HS Assay Kit (Invitrogen Q32854) and quality was checked on 1% agarose gel containing ethidium bromide at a final concentration of 0.5 mg ml^−1^. The draft genome was sequenced by MicrobesNG (Birmingham, UK) as outlined in: https://microbesng.uk/microbesng-faq/. In brief, libraries were sequenced on the Illumina MiSeq using 2 × 250 bp paired‐end protocol. Reads were trimmed using Trimmomatic v0.36 (Bolger et al., 2014) and quality checked using custom scripts and Samtools (Li et al., 2009), BedTools (Quinlan & Hall, 2010), and BWA mem (Li & Durbin, 2009). A total of 203,447 quality‐checked short reads corresponding to a mean coverage of 49.89× was obtained. The Oxford Nanopore Technologies (ONT) MinION platform was then used to close the draft genome. DNA for sequencing on the ONT platform was cleaned using the magnetic bead purification kit AMPure XP (Beckman Coulter A63880). Approximately 595 ng of DNA were prepared using SQK‐RAD004 library prep kit and loaded into the flow cell (Oxford Nanopore Technologies FLO‐MIN106). A run of 23 h produced 16.7 GB of raw reads that, after base calling using Albacore v2.0.2, produced 375,894 sequences with a maximum length of 38,637 bp and a mean of 1587.8 bp. Subsequently, hybrid assembly using Illumina‐generated short reads and MinION‐generated long reads was performed using Unicycler v0.4.4 (Wick et al., 2017). Genome completeness was manually checked using Geneious® 7.1.9 (Kearse et al., 2012) and by CheckM using 334 marker genes (Parks et al., 2015).

### Genome annotation and analysis

The genome was deposited in Genbank with accession number NZ_CP026530, and re‐annotated using the NCBI Prokaryotic Genome Annotation Pipeline (Tatusova et al., 2016). The complete genome was uploaded and analysed with the RAST (Aziz et al., 2008) (accession numbers: 598659.35), IMG/ER (Chen et al., 2017), PATRIC (Davis et al., 2020) and BlastKOALA (Kanehisa et al., 2016) annotation servers. Selected putative protein‐encoding genes were further investigated by BLASTP, searching the NCBI non‐redundant protein database (Altschul et al., 1990). The average nucleotide identity (ANI) was calculated using the calculator at https://www.ezbiocloud.net/tools. Genomic islands (Gis) were predicted by IslandViewer 4 (Bertelli et al., 2017). Phage analysis was obtained using PHASTER (Arndt et al., 2016).

### Protein extraction and analysis

Proteomics experiments were performed in two biological replicates and two technical replicates each at Rutgers University's Mass Spectrometry Facility. The protein fraction was extracted from cell biomass using the following method: 50 μl of 2X Laemmli buffer was added to each sample, which was then sonicated, heated at 60°C for 10 min, and centrifuged, with supernatant saved. The 50 μl of 8 M urea was added, sonicated and the supernatant from the previous step added back. Sample was then centrifuged and supernatant saved. The 100 μl of 2X Laemmli buffer was added, then the sample was sonicated, frozen, thawed and frozen at −80°C overnight. The supernatant from the previous step was added back, the sample was centrifuged, and protein concentration measured at 660 nm using a colorimetric assay (Pierce™ 660 nm Protein Assay Reagent; Thermofisher, Waltham, MA). SDS‐PAGE gel purification was then performed and protein bands were cut out of the gel, washed and digested with trypsin (Promega). Tryptic peptides were extracted, concentrated and desalted on a precolumn cartridge (300 μm i.d., 5 mm Dionex). A separating column (75 μm i.d., 150 mm, Nanoseparations) was used to eluate peptides. Label‐free LC–MS spectral counting of the six samples was carried out on a liquid chromatography tandem mass spectrometry using a nanoelectrospray ion source. The resulting tandem mass spectra were searched against the predicted peptide sequences encoded by the genome of *Nautilia* sp. strain PV‐1, using the open‐source software X!TandemPipeline version 3.4.3 (Langella et al., 2017) and default parameter. Only proteins identified with at least two unique peptides and with a peptide coverage >40% were used for the successive statistical analysis. Despite the different growth rates in the HPC, LPC, and LPB conditions, there was not a direct correlation between protein expression and growth performance. In proteomic analyses, a bias towards the most abundant proteins can affect results. This is particularly true for catalytic enzymes that are usually expressed at very low levels (Table S1) or for enzymes involved in DNA metabolism (e.g. replication and repair). To minimize this bias while investigating differentially expressed proteins in the different conditions, we tested two different statistical approaches to identify differentially regulated proteins: DESeq2 (Love et al., 2014.) and Qspec version 1.2.2 (Choi et al., 2008; Choi et al., 2015). As described by Langley and Mayr (2015), these two softwares use different methods to calculate differential expression: QSpec is based on a hierarchical Bayes estimation of generalized linear mixed effects model (Choi et al., 2008), whereas DESeq2, implemented in R within the Bioconductor package, uses a Benjamini–Hochberg FDR correction (Benjamini and Hochberg, 1995; Love et al., 2014). Only proteins with a corrected *p* value (*p* < 0.1) for DESeq2 and a false discovery rate (fdr) < 0.01 for QSpec were considered statistically differentially expressed between conditions. The Psort software was used to predict the protein location within the different cell compartments (Gardy et al., 2003; Nakai & Horton, 1999; Yu et al., 2010). Raw proteomic data were deposited into the ProteomeXchange database with accession number PXD022895.

## RESULTS AND DISCUSSION

### Enrichment, isolation and phylogenetic characterization of strain PV‐1

**FIGURE 1:**
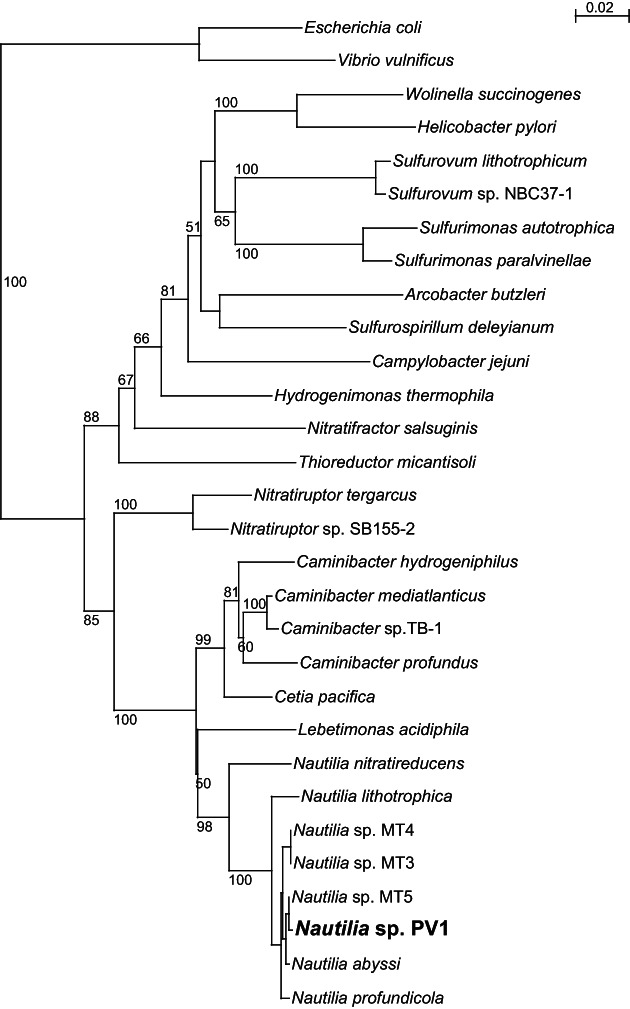
Maximum likelihood phylogenetic tree derived from 16 S rRNA gene sequences showing the position of *Nautilia* sp. strain PV‐1 within the Campylobacterota. Bootstrap values higher than 50% are based on 1000 replicates and are shown at each node. Bar, 0.02% substitutions per position. Sequences belonging to the Gammaproteobacteria were used as the outgroup. [Corrections added on 19 November 2022, after first online publication: italicization in Figure 1 has been corrected in this version.]

*Nautilia* sp. strain PV‐1 was isolated from an enrichment obtained from hydrothermal fluids released from an active vent located on the East Pacific Rise (EPR) at 9 °N (Foustoukos, 2015). The sample was collected using isobaric gas‐tight samplers (Seewald et al., 2002) and transferred directly to a shipboard continuous culture system, maintaining a constant pressure of 25 MPa throughout the procedure (Foustoukos & Pérez‐Rodríguez, 2015). The primary enrichment was carried out in the shipboard continuous culture system at 50°C and 25 MPa supplied with a constant flow of H_2_/HCO_3_^−^/NO_3_^−^‐enriched media (1.3, ~7, and ~5 mM, respectively) (Foustoukos, 2015). The pure culture was subsequently isolated by a series of end‐point dilutions carried out at 55°C, 0.2 MPa in batch cultures under a H_2_/CO_2_ (80:20) headspace. In line with other members of the family *Nautiliaceae*, strain PV‐1 is a chemolithoautotroph that conserves energy by coupling the oxidation of H_2_ to the reduction of NO_3_ or S^0^. Phylogenetic analysis of the 16 S rRNA gene of the resulting pure culture placed strain PV‐1 in a cluster of sequences derived from *Nautilia* spp. isolated from the EPR, including three uncharacterized strains (*Nautilia* sp. MT‐3, MT‐4 and MT‐5; Voordeckers et al., 2008; Figure 1). Identification using the 16 S rRNA gene sequence (https://www.ezbiocloud.net/identify) indicated that the closest relatives to strain PV‐1 were *Nautilia abyssi* strain PH1209 (sequence identity: 99.3%; Alain et al., 2009) and *N. profundicola* strain AmH (sequence identity: 99.1%; Smith et al., 2008).

### Overview of the genome of strain PV‐1

The genome of strain PV‐1 contains 1,791,100 bp encoding the genes for carbon fixation via the reductive tricarboxylic acid cycle (rTCA cycle), consistent with its chemolithoautotrophic metabolism and that of its close relatives (Alain et al., 2009; Campbell et al., 2009). The ANI between the genomes of strain PV‐1 and *N. profundicola* indicated a similarity 81.59%. Similar to the two Campylobacteria, *Sulfurimonas denitrificans* and *N. profundicola*, strain PV‐1 encodes two putative fumarate reductase/succinate dehydrogenase (Fdr/Sdh) complexes (C3L23_RS05740–RS05755, C3L23_RS05345–RS05355), an ATP‐citrate (pro‐S‐)‐lyase (C3L23_RS03675–RS03680), and a pyruvate:ferredoxin oxidoreductase (C3L23_RS01200–RS01215). As expected, all the genes involved in the pentose phosphate pathway as well as the genes necessary for gluconeogenesis were detected (Table S1).

**FIGURE 2:**
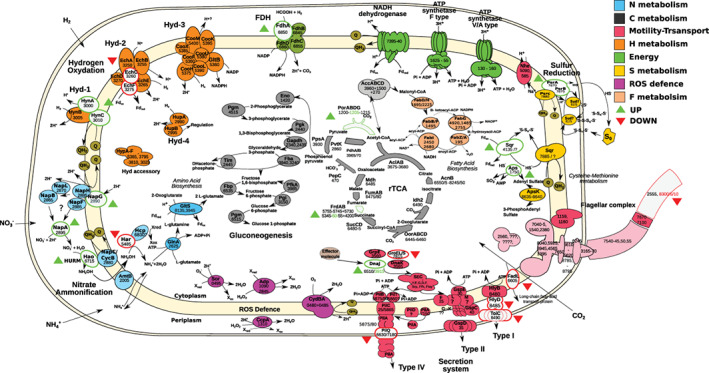
Metaproteogenomic reconstruction of the central metabolism of *Nautilia* sp.strain PV‐1. Enzyme names are reported along with the corresponding protein numbers as discussed in the text and reported in Table S1. Within each enzymatic complex, significantly (false discovery rate [fdr] < 0.01 for QSpec) overexpressed and underexpressed enzymes (outlined shapes) in the HPC versus LPC comparison are indicated by the upward green and downward red arrows, respectively. Abbreviations: Nitrate ammonification: AmtB, ammonia transporter; GlnA, glutamine synthetase; GltS, glutamate synthase; Hao, hydroxylamine:ubiquinone reductase; Har, hydroxylamine reductase; Hcp, putative iron‐sulfur cluster‐binding protein; NapABCFGHL, periplasmic nitrate reductase complex. Hydrogen oxidation: CooFHKLMUX, carbon monoxide‐induced hydrogenase complex; EchABCEF, Ech membrane bound hydrogenase complex, ferredoxin reduction; FdhABC, formate dehydrogenase; GltB, glutamate synthase; HupAB, cytosolic uptake/hydrogen sensing hydrogenase; HynABC, Ni‐Fe membrane‐bound hydrogenase; HypAF, hydrogenases expression/synthesis accessory proteins; HynABC, quinone‐reactive hydrogenase. Energy conservation: Aps, sulfate adenylyltransferase; NADH dehydrogenase and ATP synthetase are reported without the names of the single units; Nhe, sodium/hydrogen symporter. Sulfur reduction: PsrABC, polysulfide oxidoreductase complex; Sqr, sulfide:quinone oxidoreductase; Sud, putative rhodanese‐like domain‐containing protein. Flagellar complex: for simplicity single unit names are not reported. Reductive citric acid cycle: AclAB, ATP‐citrate lyase; AcnB, aconitate hydratase; AccABCD, acetyl‐coenzyme transferase complex; FumAB, fumarate hydratase; FrdAB, fumarate reductase; Idh2, isocitrate dehydrogenase/2‐oxoglutarate carboxylase; Mdh, malate dehydrogenase; OorABCD, 2‐oxoglutarate synthase; PorABDG, pyruvate synthase; PdhAB, pyruvate dehydrogenase; PpsA, phosphoenolpyruvate synthase; PvtK, pyruvate kinase; PepC, phosphoenolpyruvate carboxylase/kinase; SucCD, succinyl‐CoA synthetase. Gluconeogenesis: Eno, enolase; Fba, fructose‐bisphosphate aldolase; Fbp, fructose‐1,6‐bisphosphatase; Gapdh, glyceraldehyde 3‐phosphate dehydrogenase; Pgm, phosphoglycerate mutase; Pgk, phosphoglycerate kinase; Pgi, phosphoglucose isomerase; Pgm, Phosphoglucomutase; Tim, triosephosphate isomerase. Biosynthesis: FabABDFGHIZ: fatty acid biosynthesis pathway. Reactive oxygen species detoxification: Adp, rubredoxin/hydroperoxide reductase; CydBA, cytochrome d ubiquinol oxidase; CcpA, cytochrome c551 peroxidase; Sor, superoxide reductase. Stress protection: DnaJK, molecular chaperone; GrpE: heat‐shock protein; GroEL/S: heat‐shock protein 60 family. Secretion systems (SEC): GspCDEFLM, type II secretion system; FadL, long‐chain fatty acid transport protein; HlyBD, ABC transporter multidrug efflux pump/type I family secretion protein; PilABCDQT, type IV secretion system/fimbrial assembly; TolC, outer membrane efflux protein

Based on genomic reconstruction, the first step of the respiratory nitrate ammonification pathway of strain PV‐1 (nitrate reduction to nitrite) is mediated by the periplasmic Nap complex (Figure 2; C3L23_RS02880–RS02895; Vetriani et al., 2014), while nitrite is subsequently converted to hydroxylamine by a periplasmic hydroxylamine:ubiquinone reductase module (C3L23_RS06715) working in reverse (Figure 2; reverse‐HURM pathway; Hanson et al., 2013). The hydroxylamine is subsequently transferred to the cytoplasm and reduced to ammonium by a cytoplasmic hydroxylamine reductase (C3L23_RS05485; Campbell et al., 2009; Hanson et al., 2013).

The genome of strain PV‐1 encodes the genes putatively involved in the respiratory reduction of elemental sulfur/polysulfides: a putative membrane‐bound polysulfide reductase (PsrABC; C3L23_RS07870–RS07880), whose role in respiratory sulfur reduction was experimentally demonstrated in the Campylobacterium, *Wolinella succinogenes*; a putative periplasmic polysulfide‐binding protein (Sud; C3L23_RS09175) that provides sulfur and polysulfide to PsrABC (Klimmek et al., 1998) and an assimilatory NAD(P)H:polysulfide oxidoreductase (C3L23_RS04935) that can reduce elemental sulfur to sulfide for assimilation via cysteine and methionine synthesis (Figure 2; Campbell et al., 2009).

Strain PV‐1 also encodes two putative sulfide:quinone oxidoreductase (SQR; C3L23_RS04135 and RS07885), whose role is not completely clear. In the sulfur‐oxidizing and sulfur‐reducing Campylobacterium, *Sulfurovum* sp. NBC37‐1, SQR is believed to work in both the oxidative and reductive direction (Yamamoto et al., 2010), although an involvement of this enzyme in the detoxification of sulfide was recently postulated in the thermophilic anaerobe, *Thermovibrio ammonificans* (Aquificae; Jelen et al., 2018). Of the two SQR‐coding genes present in the genome of strain PV‐1, one (C3L23_RS07885) is adjacent to the subunit C of the genes encoding the polysulfide reductase (PsrABC), and its product is predicted to be periplasmic by the software Psort (Yu et al., 2010). However, the second SQR‐coding gene of strain PV‐1 (C3L23_RS04135) is not associated with other genes involved in sulfur metabolism and is predicted to be cytoplasmic. The putative different cellular localizations of the two SQR imply distinct roles. Because of its unclear function and to determine its role in respiratory metabolism and/or sulfide detoxification, SQR should be a high‐priority target in further biochemical, structural and gene expression studies.

In line with its hydrogenotrophic metabolism, strain PV‐1 encodes multiple hydrogenase complexes. Of the four annotated NiFe‐hydrogenases, the primary H_2_‐oxidizing enzyme is thought to be Hyd‐1, a three‐subunit membrane‐bound hydrogenase that reduces quinone (menaquinone). Hyd‐1 is predicted to be a NiFeSe‐enzyme whose activity depends on selenium (Se). Overall, the hydrogenases encoded by strain PV‐1 include a periplasmic (C3L23_RS02955–RS02960; Table S1) and a membrane associated (C3L23_RS03000–RS03010) uptake [Ni‐Fe] hydrogenase, a quinone‐reactive hydrogenase (Hyn; Hyd‐1 in Figure 2), a cytoplasmic hydrogen‐sensing hydrogenase (Hyd‐4; C3L23_RS02990–RS02995), and two enzymes predicted as hydrogen‐evolving hydrogenases of Group 4: Ech (Hyd‐2 in Figure 2; C3L23_RS03250–RS03280) and Hyc (C3L23_RS03855–RS03880; Table S1). In addition, Hyd‐3 (C3L23_RS05375–RS05395; Figure 2), an eight‐subunit enzyme, is related to the CO‐induced hydrogenases. However, the genome of strain PV‐1 does not encode a canonical CO‐oxidizing enzyme. The redundancy of different types of hydrogenases reflects the importance of hydrogen metabolism in this strictly hydrogenotrophic bacterium, a metabolic characteristic that is shared among all members of the family Nautiliaceae. However, the physiological role of each of these enzymes has not yet been elucidated in these bacteria (Campbell et al., 2009), and experiments addressing the interplay between H_2_, CO_2_ and Se availability will shed light on the activity of hydrogenases and their adaptation to pressure and trace metal limitations.

The genome of strain PV‐1 also encodes a four‐subunit formate dehydrogenase (FDH; Figure 2; C3L23_RS06850–RS06860). At atmospheric pressure and otherwise optimal conditions, strain PV‐1 does not grow with 20 mM formate as the electron donor under an N_2_/CO_2_ (80:20, 200 kPa) headspace, conditions that support growth of *N. profundicola* (Campbell et al., 2009; Hanson et al., 2013). However, in a pure H_2_ headspace, the same concentration of formate supports growth, albeit reduced compared to CO_2_, indicating that strain PV‐1 can use formate as a carbon source. The iron–sulfur catalytic subunit of the formate dehydrogenase reduces NAD(P) + at the expense of formate and CO_2_ production, which is then likely harnessed by the rTCA enzymes for fixation.

**FIGURE 3:**
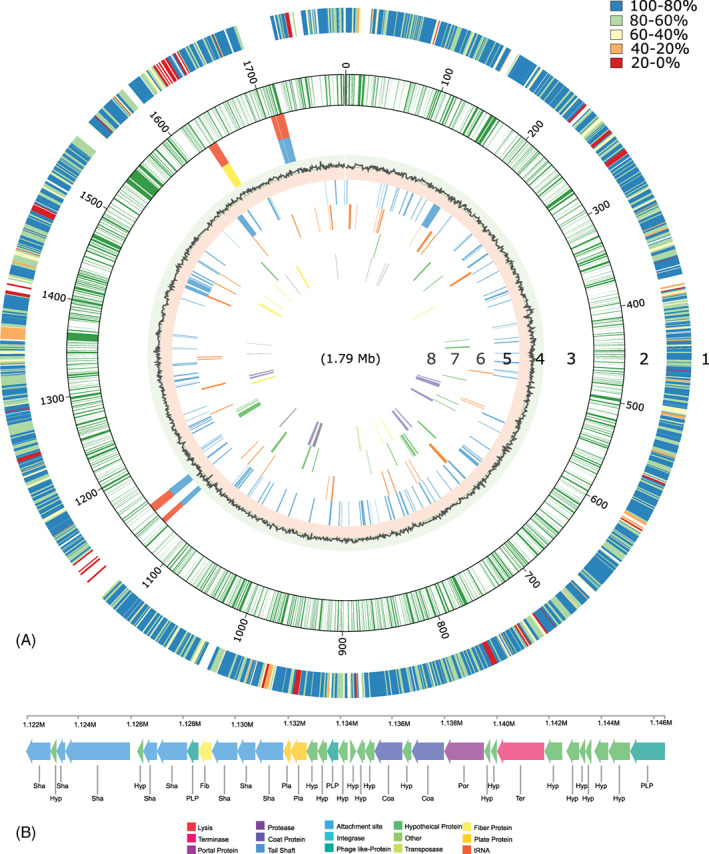
(A) Genome structures of *Nautilia* sp. strain PV‐1. Features, starting with the outermost circle: 1. Comparative amino acid percent identity between *Nautilia* sp. strain PV‐1 and *Nautilia profundicola* strain Amh; 2. Gene distribution within PV‐1 genome; 3. Predicted genomic islands in the genome of strain PV‐1 by IslandPath‐DIMOB (blue), SIGI‐HMM (yellow); predicted by all tools within IslandViewer (orange); 4. GC skew; 5. Genetic information processing (blue lines); 6. Membrane transport (orange lines); 7. Carbon metabolism: glycolysis/gluconeogenesis reductive citric cycle (green lines); 8. Energy metabolism: hydrogenase (grey lines) nitrogen metabolism (purple lines) sulfur metabolism (yellow lines). (B) Complete genome of the prophage of *Nautilia* sp. strain PV‐1

Two regions of the genome of strain PV‐1 were initially predicted as possible genomic islands (using IslandViewer 4; Bertelli et al., 2017; Figure 3A); further analysis confirmed the first region to be a complete bacteriophage (using PHASTER; Arndt et al., 2016; Figure 3B), whereas the second region, although containing a putative bacteriophage integrase (C3L23_09105) and a phage‐associated RNA ligase (C3L23_RS09100), was not annotated as a complete phage (Table S1). The closest relatives to strain PV‐1, *N. abyssi* and *N. profundicola*, do not host complete prophages (Campbell et al., 2009). The ecological and evolutionary impact of viral populations at hydrothermal vents has been inferred mainly from metagenomic and metatranscriptomic sequence data (Labonté et al., 2019), while few virus/host systems have been isolated and studied in detail. One such relevant example is phage NrS‐1, which infects the vent Campylobacterium, *Nitratiruptor* sp. strain SB155‐2. Investigation of phage NrS‐1, along with phages of pathogenic Campylobacterota, suggests that these phages may have been acquired early in the evolution of their bacterial hosts (Clark & Ng, 2008; Yoshida‐Takashima et al., 2013).

### Physiological characterization and growth rate estimation of PV‐1 at high and low pressure

To investigate the response of strain PV‐1 to in situ seafloor pressure (i.e. 20 MPa), we conducted a series of continuous culture experiments utilizing a high‐pressure–high‐temperature chemostat (Foustoukos & Pérez‐Rodríguez, 2015; Houghton et al., 2019). Experiments were conducted at 55°C and at pressures ranging from atmospheric to 20 MPa, with carbon dioxide as the carbon source and hydrogen and nitrate as the electron donor and acceptor, respectively. The use of an open culture system allowed the control of pressure, temperature and flow rate (which controls nutrient availability) inside the chemostat vessel to approximate as closely as possible the in situ conditions of growth (Foustoukos, 2015). Compared to batch cultures, where the concentration of cells increases during the exponential phase of growth as carbon dioxide, hydrogen, and nitrate decrease, the chemostat allows the population to reach a steady state where the concentrations of cells, carbon and electron donor/acceptors remain constant. However, in the chemostat, increasing or decreasing the flow rate can modulate the absolute concentrations of carbon and electron donor/acceptor according to the fundamental concepts of continuous culturing and, thus, control microbial growth inside the chemostat (Herbert et al., 1956). This allows the maintenance of cultures at a steady‐state cell density to collect biomass samples large enough for quantitative molecular analysis. Under steady‐state conditions for biomass growth, the dilution rate (*D*) is equal to the specific growth rate (*μ*). Complete wash‐out of the cultured microorganisms from the chemostat occurs when *D* is greater than the maximum specific growth rate (μ_max_). The dilution rate is defined as the ratio between flow rate and the volume of the chemostat (Herbert et al., 1956).

**FIGURE 4:**
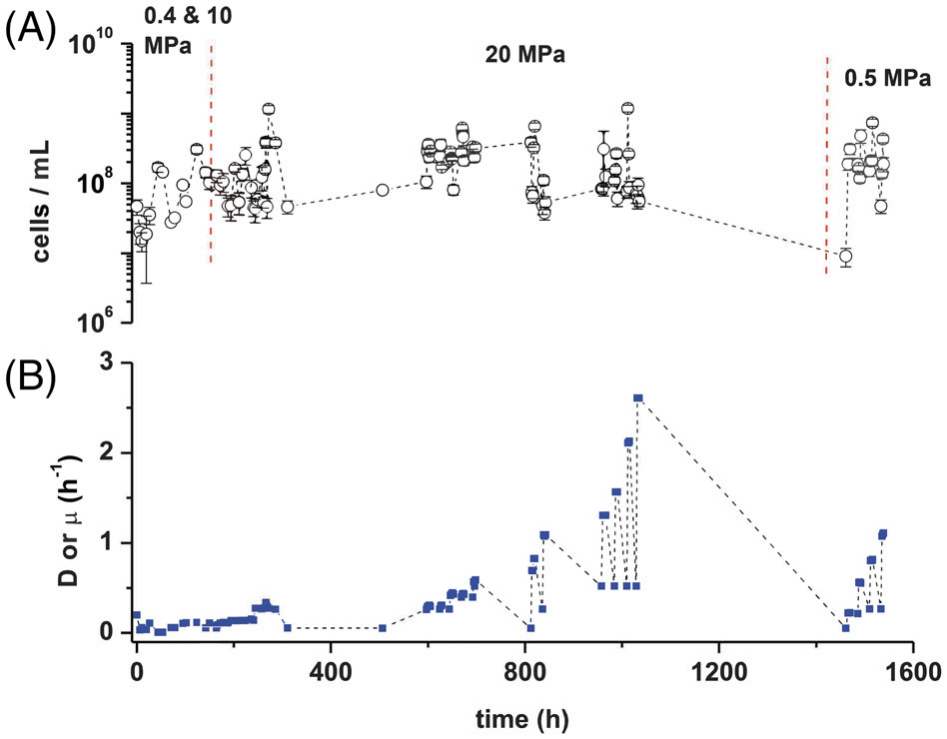
Variation of growth parameters of *Nautilia* sp. strain PV‐1 over the course of the 1633‐hour experiment in the chemostat at different pressures. (A) Cell concentration over time. (B) Dilution rate/growth rate (D or μ) over time. The different pressure regimes are indicated at the top of the graphs.

In the course of a 1633‐h long experiment at different pressures (0.4–20 Megapascals, MPa) and dilution rates (0.06–2.61 h^−1^), we monitored the growth and metabolic activity of strain PV‐1 (Figure 4; Table S2). While strain PV‐1 could grow at all tested pressures (up to 20 MPa), we carried out growth rate estimation experiments at 0.5 and 20 MPa by constraining the transition from steady‐state cultures to washout conditions (Coates et al., 1996; Contois, 1959; Herbert et al., 1956; Wood & Kelly, 1981). Overall, cultures were maintained at steady‐state condition within the range of dilution rates adopted (Table S2). The maximum dilution rates at which PV‐1 sustained growth at 0.5 and 20 MPa were 1.60 and 2.61 h^−1^, respectively (Figure 4). These findings indicate that, within the 0.5–20 MPa pressure range, strain PV‐1 grew more efficiently at 20 MPa (doubling time of ~16 min at 20 MPa and 55°C) and therefore is a thermopiezophile. The attained growth rate at 20 MPa is in agreement with the flow rate of hydrothermal fluids measured in the vicinity of the vent site from which strain PV‐1 was isolated (Germanovich et al., 2015).

**FIGURE 5:**
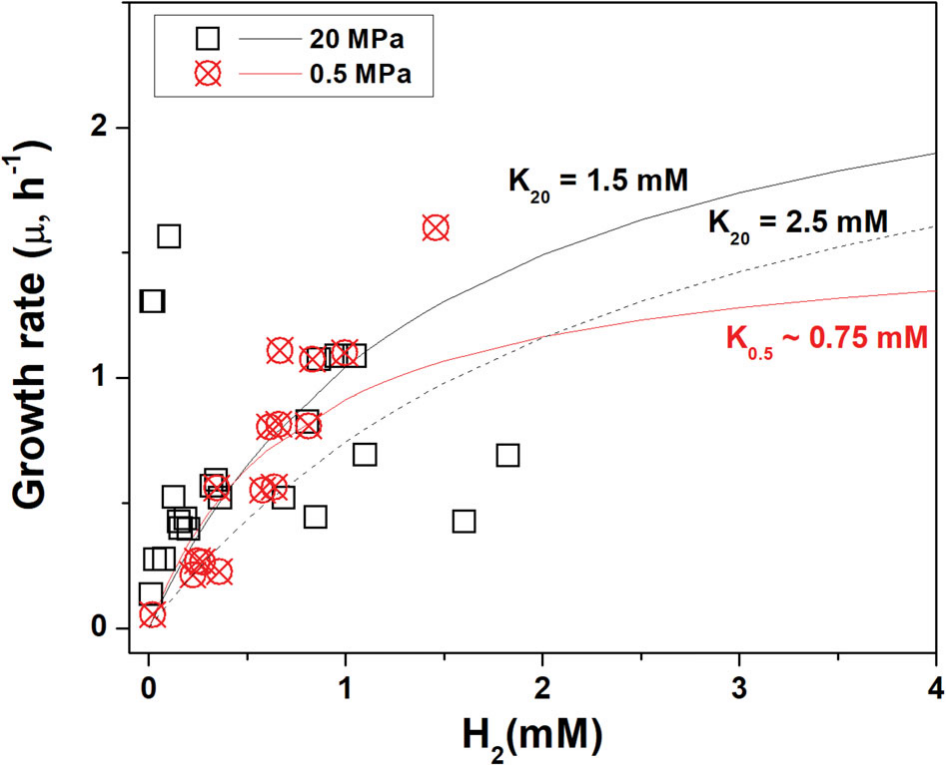
Variation of grow rate of *Nautilia* sp. strain PV‐1 in relation to the concentration of dissolved hydrogen at 0.5 and 20 MPa

We further examined the protein expression profiles of strain PV‐1 under three conditions: (1) high pressure in the chemostat (HPC; time: 838–842 h; Table S2); (2) low pressure in the chemostat (LPC; time: 1460–1633 h; Table S2); and (3) low pressure in batch cultures (LPB). Analyses of the concentration of dissolved carbon dioxide, hydrogen and nitrate in the source medium and in the outflow of the chemostat indicated that hydrogen are the limiting factor (Figure 5). This was particularly relevant in the LPC condition, where the lower growth rate of strain PV‐1 required a corresponding low flow rate to allow for sufficient residence time and avoid cell washout. For this reason, the LPC condition should be considered as nutrient depleted compared to the HPC and LPB conditions. These caveats of the experimental design were taken into account in the interpretation of the proteomic data.

### Overview of proteomic analysis

To assess the adaptation of strain PV‐1 to different pressure regimes, we analysed its proteomic profiles at 20 MPa (HPC condition) and 0.5 MPa (LPC condition) in the chemostat at a flow rate of 2 and 0.2 ml min^−1^, respectively. The choice of the flow rates was dictated by the specific behaviour of strain PV‐1 at the different pressure regimes: at 20 MPa, the growth rate of strain PV‐1 was 1.1 h ^−1^ at 2 ml min^−1^, whereas its growth rate of at 0.5 MPa dictated a lower flow rate of 0.2 ml min^−1^ to avoid cell washout. We also investigated protein expression of strain PV‐1 grown in batch culture at 0.2 MPa (LPB condition). The HPC condition was similar to the original shipboard enrichment of vent fluids, while the LPB condition replicated the procedure used to isolate strain PV‐1. We then compared the LPC and LPB conditions to identify possible differences in protein expression between the continuous and the batch culture systems. We carried out two biological and two technical replicates, respectively, for each experiment.

**FIGURE 6:**
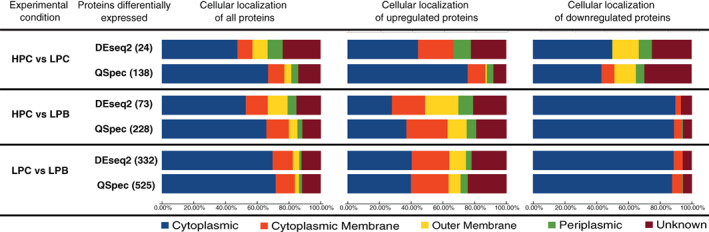
Relative abundance (%) of differentially expressed proteins by cellular localization as predicted by the DEseq2 and QSpec algorithms in the HPC versus LPC and LPC versus LPB treatments

Analysis of the proteome of strain PV‐1 (with the X!tandem pipeline, Langella et al., 2017) identified 1585 proteins that were detected at least twice. Our analysis accounted for 85% of the total predicted genes in the genome of strain PV‐1. At 20 MPa, strain PV‐1 grew faster than in any of the other conditions tested, with a doubling time of ~16 min and a growth rate of 2.6 h^−1^ (Figure 4). Despite the different growth rates in the HPC, LPC and LPB conditions, there was not a direct correlation between protein expression and growth performance. A lower number of differentially expressed proteins (24 and 138 for DEseq2 and QSpec, respectively) were identified in the HPC (20 MPa) versus LPC (0.5 MPa) comparison, whereas a higher number of differentially expressed proteins (332 and 525, respectively) were identified in the LPC versus LPB comparison (Figure 6).

Most of the differentially expressed proteins were predicted to be cytoplasmic (Figure 6). However, we found a slightly higher number of differentially expressed membrane proteins in the HPC conditions, both in HPC versus LPC (19.4% and 14.7%) and LPC versus LPB (16.8% and 14.6%) comparisons. One way to interpret this result is that membrane proteins are typically overexpressed at high pressures (Oger & Jebbar, 2010). In contrast, cytoplasmic proteins constituted the overwhelming majority of downregulated proteins in each comparison analysed (Figure 6). Interestingly, the growth in the chemostat at low pressure seems to stimulate mainly the expression of membrane‐related proteins (Figure 6; LPC vs. LPB comparison). As more detailed analyses showed, different proteins were up‐ and downregulated at high pressure (HPC vs. LPC) and at low pressure (LPC vs. LPB). For instance, proteins involved in motility and transport (e.g. flagellin C3L23_RS08305 and C3L23_RS08310) resulted overexpressed at low pressure (LPC vs. LPB) while inhibited under high pressure (HPC vs. LPC; Table S1).

In the following paragraphs, we report and discuss the expression profiles of proteins involved in central metabolic pathways, information processing, lipid metabolism, motility and transport processes, as well as phage proteins. In each paragraph, the HPC versus LPC and LPC versus LPB experimental conditions are compared, respectively.

### Energy metabolism

#### HPC versus LPC

The comparison between the HPC and the LPC experimental regimes was aimed at identifying the effect of pressure on the expression profile of strain PV‐1 under continuous culture conditions. In the HPC and LPC regimes, the concentrations of both NO_3_^−^ and of the H_2_/CO_2_ gas mix used to feed the chemostat were kept constant (0.31 MPa H_2_/CO_2_; Table S1) to minimize the effect of substrate availability and to highlight the pressure effect. When measured in the outflow, the concentration of NO_3_^−^ was found not to be limiting for growth, while the concentration of H_2_ varied in accordance with its consumption at the different growth rates (i.e. dilution rates; Figure 5).

Under high hydrostatic pressure, two subunits of the H_2_‐evolving hydrogenases *ech* (C3L23_RS03260‐C3L23_RS03275) were downregulated, while the non‐Se HynAB subunits of Hyd‐1 appeared to be upregulated (C3L23_RS03000–RS03010; Figure 2), suggesting that the concentration of Se employed in the media (2 nM SeO_3_^2−^; Vetriani et al., 2004) might be limiting. Under conditions of Se limitation, we hypothesize that its two soluble subunits (HynAB; C3L23_RS03000 and RS03005) are replaced by an adjacently encoded second set of a non‐Se AB subunits (C3L23_RS02955 and RS02960), which we predict would bind to the same membrane‐bound quinone‐reactive subunit (HynC, C3L23_RS03010; Figure 2). The Hyd‐3 multi‐subunit complex, related to the CO‐induced hydrogenases (C3L23_RS05375 ‐ RS05395), was overexpressed at high pressure (Table S1). We propose that Hyd‐3 generates NADPH to drive CO_2_ fixation by the rTCA cycle because a predicted NADPH‐binding subunit appears to be co‐expressed with the hydrogenase subunits (C3L23_RS05360). The physiological roles of the multiple hydrogenases are still not clear and may reflect an adaptation to variable hydrogen concentrations and redox regimes (Campbell et al., 2009; Laurinavichene et al., 2002). Proteomic analysis revealed that the membrane‐bound hydrogenase, Hyd‐1, associated with the quinone pool is upregulated at 20 MPa (C3L23_RS03000–RS03010) (Figure 2). Besides a cytosolic regulatory hydrogenase (Hyd‐4), strain PV‐1 contains three membrane‐bound hydrogenases that are conserved in several isolates within the *Nautiliales* (Vignais & Billoud, 2007). These membrane‐bound enzymes (Hyd‐1, 2, and 3 in Figure 2) are linked to the proton gradient across the cytoplasmic membrane and may be affected by the stress induced on membrane structure at high pressures (Jebbar et al., 2015). It is also possible that the membrane‐bound hydrogenases of strain PV‐1 are involved in controlling the leakage of H^+^ ions across the membrane. In some organisms, compatible solutes can grant some resistance to high pressure by providing enough ionic strength to push back against membrane leaking with charge gradients. We hypothesize that the Mnh antiporter of strain PV‐1 (Table S1) might be part of an ion exchange paired with H^+^ transporting hydrogenases. Overall, the observed redundancy and diversity of hydrogenases in strain PV‐1 likely represents a mechanism used to adapt to the dynamic conditions of its habitat.

**FIGURE 7:**
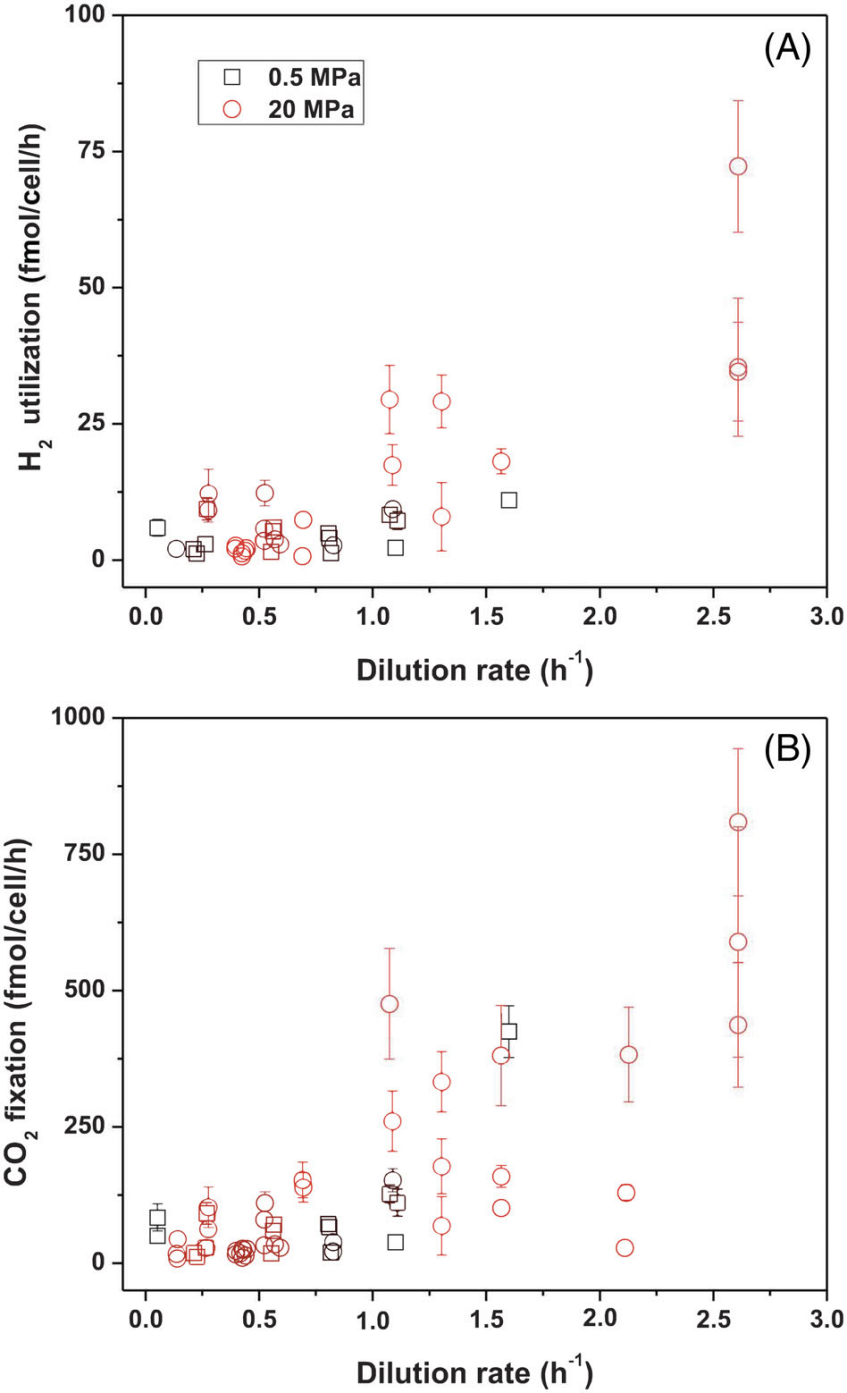
Rates of hydrogen utilization (A) and carbon fixation (B) in *Nautilia* sp. strain PV‐1 relative to the dilution rate at 0.5 and 20 MPa

While the H_2_ concentration used to feed the chemostat was kept constant (~ 2 mM H_2(aq)_ from 218 to 1633 h), H_2_ consumption varied depending on the growth rate of strain PV‐1 and the pressure conditions. Hence, the observed hydrogenase expression pattern may reflect the different hydrostatic pressure regimes, the availability of H_2_ in the chemostat, or a combination of both factors. For example, when H_2_ becomes more available, strain PV‐1 appears to respond by upregulating the Hyd‐1 hydrogenase (Figure 2). This might also be linked to the higher rates of H_2_ utilization in the HPC (20 MPa) relative to the LPC (0.5 MPa) cultures (Figure 7A). The rates of H_2_ utilization at the maximum growth stages of the HPC and LPC cultures were 72 ± 12 and 11 ± 1.2 fmol cell^−1^ h^−1^, respectively. Based on the extent of H_2_ utilization at varying growth rates (Figure 7A), the saturation constant *K*_*s*_ that describes substrate limitations (Herbert et al., 1956) is estimated to range between 1.5 and 2.5 mM at 20 MPa and less than 1 mM at 0.5 MPa (Figure 5). With *K*_*s*_ values approaching the H_2(aq)_ concentrations in the medium feed (~2 mM), we hypothesize that the maximum growth rate of strain PV‐1 at 20 MPa might be even higher than 2.6 h^−1^ under H_2_ non‐limiting conditions. Cumulatively, these findings suggest that the piezophilic behaviour of strain PV‐1 appears to be accompanied by increased energy requirements at high pressures. This is consistent with what has been observed in other experiments involving the Gammaproteobacterium, *Thiomicrospira thermophila*, where pressure at 10 MPa increased the bioenergetic demand for the electron acceptor (O_2_; Houghton et al., 2019).

One of the four subunits of the formate dehydrogenase (FdhA, C3L23_RS06845–C3L23_RS06855) was upregulated in the high‐pressure regime (C3L23_RS06850) (Figure 2) despite the absence of formate in the culture medium. The constitutive expression of the catalytic subunit of the formate dehydrogenase suggests that, under high‐pressure conditions similar to its natural habitat, strain PV‐1 might oxidize formate for extra CO_2_ access, consistent with the observation that this bacterium can utilize formate as a carbon source. Similarly, while strain PV‐1 was grown using nitrate as the sole electron acceptor and sulfur was not present in the culture medium, four different enzymes involved in sulfur respiration were found overexpressed under high pressure (Figure 2 and Table S1): two subunits of the membrane‐bound polysulfide reductase (PsrAB; C3L23_RS07870‐RS07875), the sulfide:quinone oxidoreductase (Sqr, C3L23_RS04135), and the sulfate adenylyltransferase (Aps, C3L23_RS01750). Overall, these observations show that these pathways are induced at 20 MPa and suggest that strain PV‐1 may express them constitutively in its natural habitat, where the bacterium is exposed simultaneously to multiple electron acceptors.

Two of the four subunits of the periplasmic nitrate reductase complex (NapA, and NapG, C3L23_02895 and C3L23_RS02890) were upregulated under pressure (Figure 2 and Table S1), although the concentration of nitrate in the chemostat's outflow indicates that this electron acceptor was not a limiting factor. A similar result was found in the proteomic analysis of *Photobacterium profundum* strain SS9 under pressure, probably linked to a metabolic switch toward anaerobic respiration (Le Bihan et al., 2013). Since most of the periplasmic nitrate reductase complex proteins are membrane‐associated, their observed overexpression may be due to pressure exerting stress on the cytoplasmic membrane and requiring faster turnover (Michoud & Jebbar, 2016; Oger & Jebbar, 2010).

Two key enzymes of the reverse‐HURM pathway for nitrite reduction were differentially expressed in the high‐pressure regime: the hydroxylamine oxidoreductase (Hao; C3L23_RS06715) was overexpressed and the hydroxylamine reductase (Har; C3L23_RS05485) was underexpressed (Figure 2 and Table S1). One possible interpretation of the observed contrasting expression profiles of the enzymes involved in nitrite reduction is that Hao, which is membrane‐associated, is degraded more rapidly or is less stable (i.e. it has a faster turnover rate) than Har at high pressure and therefore is overexpressed. Another possible, but not mutually exclusive, interpretation of the data is that Hao has a lower catalytic efficiency than Har at high pressure; hence, more copies of the Hao enzyme are needed to produce sufficient quantities of hydroxylamine, which is subsequently reduced to ammonium by Har.

#### LPC versus LPB

The comparison between the LPC and the LPB experimental regimes was aimed at identifying the effect of the growth conditions (continuous vs. batch) on the expression profile of strain PV‐1, while maintaining constant pressure. The doubling time and growth rate of strain PV‐1 when grown in batch were 86 min and 0.48 h^−1^, respectively, compared to a growth rate of 1.1 or 1.6 h^−1^ in the LPC regime, indicating that the growth performance of strain PV‐1 was lowest in the LPB regime. Clearly, growth was nutrient limited under LPB relative to the LPC conditions, which is consistent with the theory of continuous culturing approaches (Herbert et al., 1956). Comparing the LPC and the LPB conditions, we observed the highest numbers of differentially expressed proteins (332 and 525, identified by DESeq2 and QSpec, respectively; Figure 6).

Most of the proteins involved in sulfur respiration were down‐regulated in the LPC regime, including the polysufide reductase (PsrB), the dissimilatory sulfate adenylyltransferase (Aps) and the sulfide:quinone oxidoreductase (Sqr; Table S1).

The nitrate ammonification pathway was differentially expressed between the two low‐pressure conditions, indicating a distinctive response of strain PV‐1 to the continuous (LPC) and batch (LPB) culture regimes. In particular, the hydroxylamine oxidoreductase (Hao) was underexpressed and the hydroxylamine reductase (Har) was overexpressed in the LPC versus LPB conditions (Log2 fold changes [LFC] −0.402 and 0.918, respectively; Table S1). This is in contrast with what was observed in the HPC regime, where Hao was upregulated and Har was downregulated. Three subunits of the Nap complex, (NapALH) were all upregulated (LFC > 0.183) in the LPC condition compared to the LPB condition. In contrast, NapG, (C3L23_02900), the transmembrane Fe/S cluster, which functions as a quinone oxidoreductase and mediates the transfer of electrons from the membrane quinone pool to the NapAB periplasmic reductase complex, was overexpressed in the HPC regime (Figure 2) and underexpressed at low pressure (LFC −0.987; Table S1). Being consistent with the enhanced growth at 20 MPa (823–1035 h), the rates of NO_3_^−^ reduction inferred from the extent of extracellular NH_4_^+^ production appear to be higher relative to the low‐pressure regimes. The NH_4_^+^ concentrations in the 20 MPa cultures ranged from ~100 to 800 mM (Table S2). However, under all conditions, the d^15^N composition of the extracellular NH_4_^+^ remained constant at ~2.7 ± 1.3 ‰, which is similar to the stable N isotope fractionation (~3.5 ‰) observed in the on‐board high‐pressure incubations of the diffuse flow vent fluids from which strain PV‐1 was isolated (Foustoukos, 2015).

Overall, these findings suggest that strain PV‐1 grows faster at elevated pressure due to increased metabolic throughput, as opposed to more efficient energy generating processes.

### Carbon fixation

#### HPC versus LPC; LPC versus LPB

Strain PV‐1 can fix CO_2_ via the reductive rTCA cycle, similarly to *N. profundicola* and *Caminibacter mediatlanticus* (Campbell et al., 2009; Voordeckers et al.,  2008). Although the highest growth rate of strain PV‐1 was recorded in the chemostat under high‐pressure conditions, almost all the enzymes involved in the rTCA cycle do not seem to be affected by the variation in pressure between HPC and LPC regimes. Only one subunit of each of the pyruvate:ferredoxin oxidoreductase (C3L23_RS01205) and succinate dehydrogenase (C3L23_RS05350) were upregulated under high pressure (Figure 2).

In LPC versus LPB comparison, 27 proteins involved in central carbon metabolism (rTCA cycle and gluconeogenesis) were downregulated (LFC from −1.44 to −0.182), and only four were overexpressed (LFC from 0.22 to 1.44; Table S1).

The CO_2_ fixation rates attained by strain PV‐1 during optimal growth at 20 MPa (HPC) were 306 ± 91 fgC cell^−1^/d^−1^, while at 0.5 MPa (LPC) under the same dilution (growth) rates strain PV‐1 reached values of 37 ± 19 fgC cell^−1^ d^−1^, one order of magnitude lower (Figure 7B). CO_2_ fixation rates in the range of 20–60 fgC cell^−1^ d^−1^ have recently been reported in ambient pressure cultures of mixed microbial communities sampled from the same hydrothermal vents where strain PV‐1 originated (McNichol et al., 2022). Incubations of these samples at in situ pressures (25 MPa) have shown higher CO_2_ fixation rates (91 ± 8 fgC cell^−1^ d^−1^; NO_3_/H_2_ addition, 50°C; McNichol et al., 2018), but still lower than those attained in the high‐pressure cultures of strain PV‐1. This suggests higher growth efficiency in the pure cultures of strain PV‐1 relative to those from the EPR mixed cultures (McNichol et al., 2018).

In contrast, strain PV‐1 synthesized more copies of enzymes of the rTCA cycle in the batch culture regime (LPB), where the concentration of CO_2_ gradually decreases during growth.

The observed similar level of expression of the carbon fixation enzymes in the HPC and LPC regimes, despite the higher growth and CO_2_ fixation rates of strain PV‐1 in the HPC experiments, warrants some discussion. For instance, the expression of the ATP‐dependent citrate lyase (Acl; a key enzyme of the rTCA cycle) does not increase in the HPC regime, despite the increased growth and CO_2_ fixation rates, suggesting that individual enzymes of the rTCA cycle may operate more efficiently under elevated pressure. Considering also the expression profile of hydrogenases, we hypothesize that Hyd‐2 and Hyd‐3 provide reducing equivalents for autotrophic growth as reduced ferredoxin and NADPH, respectively (Figure 2), which would drive CO_2_ fixation by the rTCA cycle. Taken together, this would mean that the net increase of available energy (as higher H_2_ concentrations) enhances anabolism and reduces the need for careful redox balancing, resulting in an increased growth rate and differential expression. An additional aspect of this possibility is that strain PV‐1 may exhibit a stress response at low pressure, much like the facultative thermopiezophile *Marinitoga piezophila* (Alain et al., 2002; Foustoukos & Pérez‐Rodríguez, 2015). If this hypothesis is correct, cultivation under high pressure would remove the stress factor, leading to higher growth and CO_2_ fixation rates.

Overall, considering that a deep‐sea hydrothermal vent is, in its essence, a natural chemostat where carbon and energy sources are continuously supplied, the HPC experimental regime most closely mimicked the natural habitat of strain PV‐1.

### Lipid metabolism

#### HPC versus LPC

Homeoviscous adaptation allows the cell membrane lipid composition to maintain adequate membrane fluidity (Sinensky, 1974), and it is generally accepted as one of the most important responses to pressure variations (Ernst et al., 2016). Piezophilic microorganisms have been shown, through differential expression experiments and biochemical assays, to alter the viscosity of their membrane lipids (Ernst et al., 2016; Siliakus et al., 2017). To maintain the fluidity and permeability so that substrates and waste can easily diffuse across cellular membranes, piezophiles (like psychrophiles) reduce the average saturation of their membranes, which is compensated for by hydrostatic pressure forcing closer associations between individual membrane components (Ernst et al., 2016; Siliakus et al., 2017). This increased packing of lipids causes membranes to lose fluidity and permeability, transitioning to a gel phase, with an overall effect on the cell membrane similar to the exposure to low temperatures (Casadei et al., 2002; Oger & Jebbar, 2010). On the other hand, thermophiles increase the average saturation of their membranes, as the increase in temperature would normally cause membrane instability and cell lysis (Siliakus et al., 2017). This places thermopiezophiles such as strain PV‐1 in a dilemma, where conventional wisdom would dictate mutually contradictory responses within the membrane. Experimental evidence showed that, in obligate (hyper)thermopiezophiles, such as the archaea *Methanopyrus kandleri* and *Thermococcus barophilus*, the thermophilic response wins out, with highly saturated membrane lipids (Cario et al., 2015; Takai et al., 2008). In general, expression of genes related to lipid metabolism was not affected by pressure in strain PV‐1, suggesting that lipid adaptation to temperature dominates over adaptation to pressure. The only exception was a long‐chain fatty acid transport protein (C3L23_RS06605; Figure 2), which was downregulated under pressure (LFC −0.828; Table S1). This protein shares 68% identity with its homologue in *Lebetimonas natsushimae* (*Nautliaceae*; Nagata et al., 2017) and falls into the COG cluster of lipid transport and metabolism (COG2067; Marchler‐Bauer et al., 2017). The homologous enzyme in *E. coli* was described as a multifunctional integral outer‐membrane protein, required for the specific transport of exogenous long‐chain fatty acids (C12‐C18) as well as a receptor for the bacteriophage T2 (Kumar & Black, 1991). The role of this enzyme in strain PV‐1 is unknown.

#### LPC versus LPB

Proteins involved in lipid metabolism were differentially expressed when strain PV‐1 was grown at low pressure in the chemostat and in batch (Table S1). For instance, the 3‐hydroxyacyl‐[acyl‐carrier‐protein] dehydratase, FabZ form (C3L23_RS00195) was upregulated in the LPC regime (LFC 1.16). This enzyme is involved in the initiation and elongation of fatty acid synthesis, especially unsaturated fatty acids, in plants and bacteria (Heath & Rock, 1996), thereby playing an important role in the homeoviscous adaptation. Its upregulation implies an increase in membrane fluidity when PV‐1 is grown in the chemostat at low pressure, compared to the batch condition. The putative long‐chain fatty acid transport protein (C3L23_RS06605) that was downregulated at high pressure in the chemostat is upregulated at low pressure (FLC 3.586).

### Motility, transport and competence

#### HPC versus LPC; LPC versus LPB

The ferrous iron transport protein B (C3L23_RS02030) was upregulated in strain PV‐1 under pressure (0.904 LFC; Table S1). A similar trend was observed in *P. profundum* strain SS9 under high pressure (Le Bihan et al., 2013), suggesting that our results are not an experimental artefact, and is probably linked to a higher need for iron under pressure. Indeed, the overexpression of this metal ion transporter may be linked to increased requirements of the cell, perhaps because of an upregulation of other proteins that require iron as a cofactor. This finding is consistent with the upregulation of several iron‐containing enzymes in the HPC regime, for example, the Ni‐Fe hydrogenases and the formate dehydrogenase.

Membrane‐associated proteins are probably the most pressure‐sensitive biological structures, and because most of the proteins involved in motility and transport are located near or across the membrane, they were readily affected by pressure (Oger & Jebbar, 2010). Ten proteins involved in motility and transport were differentially expressed in strain PV‐1; all of these, with the exception of the glutamine ABC transporter (C3L23_RS03070) were downregulated under pressure (Table S1). The pressure‐induced upregulation of the glutamine ABC transporter in strain PV‐1 may provide the bacterium with an alternative substrate for glutamate biosynthesis. Such increased synthesis of the glutamine transporter at high pressure could also be part of a compatible solute response, as observed in several piezophiles. For example, when the piezophilic bacterium *Desulfovibrio hydrothermalis* was grown under pressure, glutamate accumulated, possibly as a protection against pressure‐induced stress (Amrani et al., 2014). Among the secretion systems of strain PV‐1, two subunits of type I (the HlyD family secretion protein and the outer membrane efflux protein C3L23_RS08485–90) and the pilus biogenesis protein PilQ of type IV (C3L23_RS06630, ‐RS07180; LFC from −0.408 to −0.422) were downregulated, while type II (general secretion pathway protein D C3L23_RS00035) was not influenced by high pressure (Figure 2 and Table S1). The bacterial secretion system is involved in several different processes: twitching motility, biofilm formation, bacteriophage infection, surface attachment, virulence, and natural transformation (Bischof et al., 2016) as well as secretion of proteins across the outer membrane of Gram‐negative bacteria (Ayers et al., 2010; Hobbs & Mattick, 1993), and bacterial DNA uptake (competence systems; Chen & Dubnau, 2004). Among the competence proteins detected in strain PV‐1, CinA (C3L23_RS03590), putatively involved in recruiting the RecA protein to the cell membrane in *Bacillus subtilis* (Kaimer & Graumann, 2010), was underexpressed in the HPC regime (LFC ‐1.67). Additionally, the competence factor ComEC (C3L23_RS05525), one of the channel proteins required for the transport of DNA into the cytosol (Dubnau & Blokesch, 2019), was overexpressed in the chemostat at low pressure (LFC 1.44; Table S1). Currently, nothing is known about competence and DNA uptake in vent Campylobacterota.

Flagellins (C3L23_RS08305‐10) were downregulated under pressure in strain PV‐1, suggesting that this bacterium relies less on flagellar motility when growing at high pressure (Table S1). In contrast, the piezophilic bacterium, *P. profundum* strain SS9, is able to utilize different flagellar systems for swimming and swarming under high‐pressure regimes (Eloe et al., 2008). In non‐piezophilic microorganisms, the flagellum has been shown to be sensitive to pressure (Bartlett, 2002). For instance, upon exposure to pressure, *E. coli* blocks the synthesis of new flagella and the functioning of previously assembled filaments (Meganathan & Marquis, 1973).

When comparing the LPC with the LPB treatments, several proteins involved in motility and transport were actively expressed in the chemostat compared to the batch condition. Of 17 differentially expressed proteins, 16 were overexpressed in the LPC experiments (LFC >0.38), and only the signal transduction histidine kinase CheA (C3L23_RS05175) was downregulated (−1.168 LFC; Table S1). Apparently, most of the functions in which these proteins are involved (e.g. motility, biofilm formation, bacteriophage infection, surface attachment, virulence, and DNA uptake) are stimulated in the LPC experiments and they are inhibited in the batch culture.

### Phage proteins

#### HPC versus LPC; LPC versus LPB

A complete prophage was found in the genome of strain PV‐1 (Figure 7B). Three phage proteins were downregulated under pressure: the putative capsid protein (C3L23_RS06090; −1.673 LFC), the phage baseplate assembly protein J (C3L23_RS06040; −2.048 LFC) and the phage tail sheath monomer (C3L23_RS06015; −0.846 LFC; Table S1). Although very little is known about the induction of the lytic cycle in prophages associated with deep‐sea bacteria, it is worth noting that the long‐chain fatty acid transport protein (C3L23_RS06605), identified as a putative receptor for bacteriophage in *Escherichia coli* (Kumar & Black, 1991), was also downregulated in strain PV‐1 under high pressure (Table S1). In contrast, these phage proteins were upregulated in the LPC experiments (with LFC >0.8). Overall, these findings suggest that phage proteins are more actively synthesized when strain PV‐1 is grown in the chemostat under low pressure. The underexpression of phage proteins at the high‐pressure condition (HPC) might be related to a stable supply of energy and carbon in this regime. However, to keep the pressure at nearly ambient values (0.5 MPa) in the LPC experiments, the gas flow rate was maintained at low levels, resulting in a limiting hydrogen supply. Such energy limitation in the LPC regime might have triggered the phage lytic cycle, which would explain the higher expression of phage proteins observed in the LPC regime.

### Transcription, replication and chaperones

#### HPC versus LPC

The DNA‐directed RNA polymerase beta subunit (C3L23_RS00890) was found slightly overexpressed (LFC 0.162) under pressure, along with the DNA mismatch repair protein, MutS (C3L23_RS03750; LFC 0.835) and the chaperone, DnaJ (C3L23_RS03915; LFC 0.731). Conversely, the replicative DNA helicase, DnaB, (C3L23_RS06160) and the heat shock chaperone GroEL (C3L23_RS07815) were downregulated (−1.182 and − 0.423 LFC, respectively; Table S1) at high pressure. The overexpression of the RNA polymerase could reflect a higher transcription rate, in line with the higher growth rate of strain PV‐1 at high pressure. However, the downregulation of the replicative DNA helicase, DnaB, at higher pressure contradicts the higher growth rate/cell division, when replication should proceed faster. One possible interpretation is that catalytic activity of DnaB is enhanced at high pressure, implying a slower protein turnover. The different expression profiles of the two chaperones (i.e. the overexpression of DnaJ and underexpression of GroEL) are more difficult to explain. Previous work based on metatranscriptomic analysis linked the overexpression of chaperones to pressure‐related adaptations of microorganisms. Pressure‐sensitive organisms as *E. coli*, *Saccharomyces cerevisiae* and the hyperthermophilic archaeon, *Thermococcus kodakarensis*, overexpress these proteins to compensate stress related to high pressure (Aertsen & Michiels, 2005; Miura et al., 2006; Vannier et al., 2015). In contrast, the psychrophilic piezophile, *P. profundum* strain SS9, and the hyperthermophilic and piezophilic archaeon, *Thermococcus barophilus*, overexpress these proteins in response to low‐pressure‐induced stress (Aertsen & Michiels, 2005; Miura et al., 2006; Vannier et al., 2015). Noteworthily, most of the proteins involved in DNA metabolism were detected in lower abundance compared with structural proteins. Overall, the effect of pressure on DNA metabolism remains unclear.

#### LPC versus LPB

The DNA‐directed RNA polymerase alpha and beta subunits (C3L23_RS08735 and RS00890) were found slightly overexpressed in the LPC regime (LFC 0.275 and 0.312, respectively), similar to what was found in the HPC regime, while the DNA mismatch repair protein, MutS (C3L23_RS03750; LFC −0.522) and the replicative DNA helicase (DnaB; C3L23_RS06165; LFC 1.531) showed an opposite trend in LPC versus LPB compared to the high‐pressure regime (Table S1). The chaperones, DnaJ and GroEL (C3L23_RS03915 and C3L23_RS07815) were under‐ and overexpressed, respectively, in the LPB regime (LFC 0.404 and −0.992, respectively). This is a reversal of the trend observed when strain PV‐1 was grown in the continuous high‐ and low‐pressure culture conditions and suggests that strain PV‐1 copes with different stressors depending on the culture regime by modulating the expression of chaperons accordingly.

### Translational apparatus

#### HPC versus LPC

Eleven large‐subunit and nine small‐subunit ribosomal proteins were overexpressed under pressure, together with 10 different aminoacyl tRNA synthetases (Table S1), consistent with the high growth rate of strain PV‐1 at 20 MPa. However, the overexpression of ribosomal proteins and tRNAs also suggest a higher turnover of these molecules and implies that the translational apparatus of strain PV‐1 is sensitive to high pressure. In line with this observation, ribosomes are believed to be among the most pressure‐sensitive cellular macromolecules, as pressure influences the spatial conformation of the proteins, affecting the translation activities of the ribosome–mRNA complex (Le Bihan et al., 2013; Pavlovic et al., 2005).

#### LPC vs LPB

RNA metabolism and protein synthesis were downregulated in strain PV‐1 in the chemostat at low pressure compared to batch condition, with 20 ribosomal proteins and 10 aminoacyl synthetase down‐regulated (Table S1). Opposite trends in the expression of proteins of the translational apparatus observed in the batch versus continuous cultures provide additional evidence that different culture regimes affect protein expression.

## CONCLUSIONS

In this study, we characterized the pressure adaptations of the first piezophilic *Campylobacterium* isolated from a deep‐sea hydrothermal vent, *Nautilia* sp. strain PV‐1. This bacterium has a very short doubling time at 20 MPa. Comparative proteomic analyses of strain PV‐1 grown under high‐ and low‐pressure regimes showed that most of the pressure‐induced proteins were predicted as membrane‐associated, confirming that the cell membrane is indeed affected by high pressure. Further, proteomic data revealed that, in some cases, pressure differentially affected the expression of enzymes involved in the same metabolic pathways. For instance, the hydrogenases coded by *Nautilia* sp. strain PV‐1 responded in different ways to hydrostatic pressure and/or hydrogen availability. Further studies will be necessary to elucidate the role of these hydrogenases and their response to different pressure regimes and hydrogen concentrations. Interestingly, while the maximum growth rate of strain PV‐1 was measured at 20 MPa, the enzymes of the rTCA cycle for CO_2_ fixation were not overexpressed under high pressure, suggesting that enzyme kinetics, protein stability and/or turnover rates might play a role. Overall, our work demonstrates that studies of microbial physiology and protein expression under conditions that simulate as closely as possible those found in situ are essential to better understand the physiology and ecological role of microorganisms at deep‐sea hydrothermal vents.

## AUTHOR CONTRIBUTIONS

FS participated in desinging the experiments, carried out the experimental work, analyzed the data and wrote the manuscript. DF and CV designed the experiments, supervised and participated in the experimental work, analyzed the data and wrote the manuscript. SP, KM and IS carried out the experimental work and edited the manuscript. MWA and GJS participated in data analyses and edited the manuscript. DG helped desinging the experiments and edited the manuscript.

## CONFLICT OF INTEREST

The authors have no conflict of interest to declare.

## Supporting information


**Figure S1:** Supporting Information

**Table S1** (Excel spreadsheet). Sheet 1: differentially expressed proteins, listed by functional category. Sheet 2: deseq2 algorithm statistical data. Sheet 3: qspec algorithm statistical data. Sheet 4: complete annotation of the genome and proteome of *Nautilia* sp. strain PV‐1.

**Table S2:** Physicochemical conditions of the chemostat experiment.

## Data Availability

The authors made all data publicly available. The genome of Nautilia sp. strain PV‐1 was deposited in Genbank (https://www.ncbi.nlm.nih.gov/genbank/submit/) with accession number NZ_CP026530, and in RAST (https://rast.nmpdr.org/rast.cgi) with accession numbers 598659.35. Raw proteomic data were deposited into the ProteomeXchange database with accession number PXD022895.
