# Supplementary material for: Adaptations to high pressure of Nautilia sp. strain PV‐1, a piezophilic Campylobacterium (aka Epsilonproteobacterium) isolated from a deep‐sea hydrothermal vent
Source: Environ Microbiol. 2022 Oct 31;24(12):6164–83. doi: 10.1111/1462-2920.16256 (PMC10092268; doi:10.1111/1462-2920.16256)
Supplement: Supplementary file 1 — Figure S1: Supporting Information [file EMI-24-6164-s002.pdf]

>C3L23_RS03690
TTAATGGAGAGTTTGATCCTGGCTCAGAGTGAACGCTGGCGGCATGCTTAACACATGCAAGTCGAGGGGC
AGCAGGCGGGAACTTCGGTTCCCGTGCTGGCGACCGGCGGACGGGTGAGTAACACGTAGCTACTTGCCCC
ACAGAGGGGGATAACACACCGAAAGGTGTGCTAATACCGCATACACCCGAGAGGGGAAAGGGCTTCGGTC
CGCTGTGGGATAGGGCTGCGGCGTATCAGCTAGTTGGTGAGGTAACGGCTTACCAAGGCGATGACGCGTA
GCTGGTCTGAGAGGATGATCAGCCACACTGGAACTGAGACACGGTCCAGACTCCTACGGGAGGCAGCAGT
GGGGAATATTGGGCAATGGGGGAAACCCTGACCCAGCAATGCCGCGTGGAGGAAGAAGCCTTTCGGGGTG
TAAACTCCTTTTGCAGGGGAAGAAACTGACGGTACCCTGCGAATAAGCTCCGGCTAACTCCGTGCCAGCA
GCCGCGGTAATACGGGGGGAGCGAGCGTTACTCGGAATCACTGGGCGTAAAGGGTGCGTAGGCGGTCAGA
TAAGTTGGGAGTGAAATCCTATGGCTCAACCATAGAACTGCTTCCAAAACTGTCTGACTAGAGTTCGGGA
GAGGCCAAGGGAATTCCTGGTGTAGGGGTGAAATCCGTAGAGATCAGGAGGAATGCCGAAAGCGAAGGCG
CTTGGCTGGAACGAAACTGACGCTGAGGCACGAAAGCGTGGGGAGCAAACAGGATTAGATACCCTGGTAG
TCCACGCCCTAAACGATGGGTACTAGTGGTTGGGGGGACAGTCCCCCAGTCACGCAGCAAACGCGATAAG
TACCCCGCCTGGGGAGTACGGCCGCAAGGCTAAAACTCAAAGGAATAGACGGGGACCCGAACAAGCGGTG
GAGCATGTGGTTTAATTCGAAGATACGCGAAGAACCTTACCTGGGCTTGACATCCACGGAACCCTGCAGA
GATGCGGGGGTGCTACTTCGGTAGAGCCGTGAGACAGGTGCTGCATGGCTGTCGTCAGCTCGTGTCGTGA
GATGTTGGGTTAAGTCCCGCAACGAGCGCAACCCCTGTCCTTAGTTGGCATCAGTTCGGCTGGCCACTCT
AAGGAGACTGCCCGGGCAACCGGGAGGAAGGTGGGGATGACGTCAAGTCATCATGGCCCTTATGTCCAGG
GCGACACACGTGCTACAATGGCCGGGACAGAGAGAKGCGAAACCGCGAGGTGGAGCAAATCTCTAAACCC
GGTCTCAGTTCGGATTGCACTCTGCAACTCGAGTGCATGAAGGCGGAATCGCTAGTAATCGCGGATCAGC
CATGCCGCGGTGAATACGTTCCCGGGTCTTGTACTCACCGCCCGTCACACCATGGGAGTCGGGTTCACCC
GAAGTCGGTATCCCTAAGAYAGGGGCCGCCTACGGTGGACCCGGCGACTGGGGTGAAGTCGTAACAAGGT
AGCCGTAGGAGAACCTGCGGCTGGATCACCTCCTTT
>C3L23_RS00785
TTAATGGAGAGTTTGATCCTGGCTCAGAGTGAACGCTGGCGGCATGCTTAACACATGCAAGTCGAGGGGC
AGCAGGCGGGAACTTCGGTTCCCGTGCTGGCGACCGGCGGACGGGTGAGTAACACGTAGCTACTTGCCCC
ACAGAGGGGGATAACACACCGAAAGGTGTGCTAATACCGCATACACCCGAGAGGGGAAAGGGCTTCGGTC
CGCTGTGGGATAGGGCTGCGGCGTATCAGCTAGTTGGTGAGGTAACGGCTTACCAAGGCGATGACGCGTA
GCTGGTCTGAGAGGATGATCAGCCACACTGGAACTGAGACACGGTCCAGACTCCTACGGGAGGCAGCAGT
GGGGAATATTGGGCAATGGGGGAAACCCTGACCCAGCAATGCCGCGTGGAGGAAGAAGCCTTTCGGGGTG
TAAACTCCTTTTGCAGGGGAAGAAACTGACGGTACCCTGCGAATAAGCTCCGGCTAACTCCGTGCCAGCA
GCCGCGGTAATACGGGGGGAGCGAGCGTTACTCGGAATCACTGGGCGTAAAGGGTGCGTAGGCGGTCAGA
TAAGTTGGGAGTGAAATCCTATGGCTCAACCATAGAACTGCTTCCAAAACTGTCTGACTAGAGTTCGGGA
GAGGCCAAGGGAATTCCTGGTGTAGGGGTGAAATCCGTAGAGATCAGGAGGAATGCCGAAAGCGAAGGCG
CTTGGCTGGAACGAAACTGACGCTGAGGCACGAAAGCGTGGGGAGCAAACAGGATTAGATACCCTGGTAG
TCCACGCCCTAAACGATGGGTACTAGTGGTTGGGGGGACAGTCCCCCAGTCACGCAGCAAACGCGATAAG
TACCCCGCCTGGGGAGTACGGCCGCAAGGCTAAAACTCAAAGGAATAGACGGGGACCCGAACAAGCGGTG
GAGCATGTGGTTTAATTCGAAGATACGCGAAGAACCTTACCTGGGCTTGACATCCACGGAACCCTGCAGA
GATGCGGGGGTGCTACTTCGGTAGAGCCGTGAGACAGGTGCTGCATGGCTGTCGTCAGCTCGTGTCGTGA
GATGTTGGGTTAAGTCCCGCAACGAGCGCAACCCCTGTCCTTAGTTGGCATCAGTTCGGCTGGCCACTCT
AAGGAGACTGCCCGGGCAACCGGGAGGAAGGTGGGGATGACGTCAAGTCATCATGGCCCTTATGTCCAGG
GCGACACACGTGCTACAATGGCCGGGACAGAGAGAKGCGAAACCGCGAGGTGGAGCAAATCTCTAAACCC
GGTCTCAGTTCGGATTGCACTCTGCAACTCGAGTGCATGAAGGCGGAATCGCTAGTAATCGCGGATCAGC
CATGCCGCGGTGAATACGTTCCCGGGTCTTGTACTCACCGCCCGTCACACCATGGGAGTCGGGTTCACCC
GAAGTCGGTATCCCTAAGAYAGGGGCCGCCTACGGTGGACCCGGCGACTGGGGTGAAGTCGTAACAAGGT
AGCCGTAGGAGAACCTGCGGCTGGATCACCTCCTTT
>C3L23_RS08335_(reversed)
TTAATGGAGAGTTTGATCCTGGCTCAGAGTGAACGCTGGCGGCATGCTTAACACATGCAAGTCGAGGGGC
AGCAGGCGGGAACTTCGGTTCCCGTGCTGGCGACCGGCGGACGGGTGAGTAACACGTAGCTACTTGCCCC
ACAGAGGGGGATAACACACCGAAAGGTGTGCTAATACCGCATACACCCGAGAGGGGAAAGGGCTTCGGTC

CGCTGTGGGATAGGGCTGCGGCGTATCAGCTAGTTGGTGAGGTAACGGCTTACCAAGGCTATGACGCGTA
GCTGGTCTGAGAGGATGATCAGCCACACTGGAACTGAGACACGGTCCAGACTCCTACGGGAGGCAGCAGT
GGGGAATATTGGGCAATGGGGGAAACCCTGACCCAGCAATGCCGCGTGGAGGAAGAAGCCTTTCGGGGTG
TAAACTCCTTTTGCAGGGGAAGAAACTGACGGTACCCTGCGAATAAGCTCCGGCTAACTCCGTGCCAGCA
GCCGCGGTAATACGGGGGGAGCGAGCGTTACTCGGAATCACTGGGCGTAAAGGGTGCGTAGGCGGTTTGA
TAAGTTGGGAGTGAAATCCTATGGCTCAACCATAGAACTGCTTCCAAAACTGTCAGACTAGAGTTCGGGA
GAGGCCAAGGGAATTCCTGGTGTAGGGGTGAAATCCGTAGAGATCAGGAGGAATGCCGAAAGCGAAGGCG
CTTGGCTGGAACGAAACTGACGCTGAGGCACGAAAGCGTGGGGAGCAAACAGGATTAGATACCCTGGTAG
TCCACGCCCTAAACGATGGGTACTAGTGGTTGGGGGGACAGTCCCCCAGTCACGCAGCAAACGCGATAAG
TACCCCGCCTGGGGAGTACGGCCGCAAGGCTAAAACTCAAAGGAATAGACGGGGACCCGAACAAGCGGTG
GAGCATGTGGTTTAATTCGAAGATACGCGAAGAACCTTACCTGGGCTTGACATCCACGGAACCCTGCAGA
GATGCGGGGGTGCTACTTCGGTAGAGCCGTGAGACAGGTGCTGCATGGCTGTCGTCAGCTCGTGTCGTGA
GATGTTGGGTTAAGTCCCGCAACGAGCGCAACCCCTGTCCTTAGTTGGCATCAGTTCGGCTGGCCACTCT
AAGGAGACTGCCCGGGCAACCGGGAGGAAGGTGGGGATGACGTCAAGTCATCATGGCCCTTATGTCCAGG
GCGACACACGTGCTACAATGGCCGGGACAGAGAGATGCGAAACCGCGAGGTGGAGCAAATCTCTAAACCC
GGTCTCAGTTCGGATTGCACTCTGCAACTCGAGTGCATGAAGGCGGAATCGCTAGTAATCGCGGATCAGC
CATGCCGCGGTGAATACGTTCCCGGGTCTTGTACTCACCGCCCGTCACACCATGGGAGTCGGGTTCACCC
GAAGTCGGTATCCCTAAGACAGGGGCCGCCTACGGTGGACCCGGCGACTGGGGTGAAGTCGTAACAAGGT
AGCCGTAGGAGAACCTGCGGCTGGATCACCTCCTTT
>C3L23_RS09205_(reversed)
TTAATGGAGAGTTTGATCCTGGCTCAGAGTGAACGCTGGCGGCATGCTTAACACATGCAAGTCGAGGGGC
AGCAGGCGGGAACTTCGGTTCCCGTGCTGGCGACCGGCGGACGGGTGAGTAACACGTAGCTACTTGCCCC
ACAGAGGGGGATAACACACCGAAAGGTGTGCTAATACCGCATACACCCGAGAGGGGAAAGGGCTTCGGTC
CGCTGTGGGATAGGGCTGCGGCGTATCAGCTAGTTGGTGAGGTAACGGCTTACCAAGGCTATGACGCGTA
GCTGGTCTGAGAGGATGATCAGCCACACTGGAACTGAGACACGGTCCAGACTCCTACGGGAGGCAGCAGT
GGGGAATATTGGGCAATGGGGGAAACCCTGACCCAGCAATGCCGCGTGGAGGAAGAAGCCTTTCGGGGTG
TAAACTCCTTTTGCAGGGGAAGAAACTGACGGTACCCTGCGAATAAGCTCCGGCTAACTCCGTGCCAGCA
GCCGCGGTAATACGGGGGGAGCGAGCGTTACTCGGAATCACTGGGCGTAAAGGGTGCGTAGGCGGTTTGA
TAAGTTGGGAGTGAAATCCTATGGCTCAACCATAGAACTGCTTCCAAAACTGTCAGACTAGAGTTCGGGA
GAGGCCAAGGGAATTCCTGGTGTAGGGGTGAAATCCGTAGAGATCAGGAGGAATGCCGAAAGCGAAGGCG
CTTGGCTGGAACGAAACTGACGCTGAGGCACGAAAGCGTGGGGAGCAAACAGGATTAGATACCCTGGTAG
TCCACGCCCTAAACGATGGGTACTAGTGGTTGGGGGGACAGTCCCCCAGTCACGCAGCAAACGCGATAAG
TACCCCGCCTGGGGAGTACGGCCGCAAGGCTAAAACTCAAAGGAATAGACGGGGACCCGAACAAGCGGTG
GAGCATGTGGTTTAATTCGAAGATACGCGAAGAACCTTACCTGGGCTTGACATCCACGGAACCCTGCAGA
GATGCGGGGGTGCTACTTCGGTAGAGCCGTGAGACAGGTGCTGCATGGCTGTCGTCAGCTCGTGTCGTGA
GATGTTGGGTTAAGTCCCGCAACGAGCGCAACCCCTGTCCTTAGTTGGCATCAGTTCGGCTGGCCACTCT
AAGGAGACTGCCCGGGCAACCGGGAGGAAGGTGGGGATGACGTCAAGTCATCATGGCCCTTATGTCCAGG
GCGACACACGTGCTACAATGGCCGGGACAGAGAGATGCGAAACCGCGAGGTGGAGCAAATCTCTAAACCC
GGTCTCAGTTCGGATTGCACTCTGCAACTCGAGTGCATGAAGGCGGAATCGCTAGTAATCGCGGATCAGC
CATGCCGCGGTGAATACGTTCCCGGGTCTTGTACTCACCGCCCGTCACACCATGGGAGTCGGGTTCACCC
GAAGTCGGTATCCCTAAGACAGGGGCCGCCTACGGTGGACCCGGCGACTGGGGTGAAGTCGTAACAAGGT
AGCCGTAGGAGAACCTGCGGCTGGATCACCTCCTTT

Range 1: 143379 to 144884
