# Supplementary material for: Adaptations to high pressure of Nautilia sp. strain PV‐1, a piezophilic Campylobacterium (aka Epsilonproteobacterium) isolated from a deep‐sea hydrothermal vent
Source: Environ Microbiol. 2022 Oct 31;24(12):6164–83. doi: 10.1111/1462-2920.16256 (PMC10092268; doi:10.1111/1462-2920.16256)
Supplement: Supplementary file 3 — Table S2: Physicochemical conditions of the chemostat experiment. [file EMI-24-6164-s001.docx]

**Table S1:** Experimental conditions and measured concentrations from the series of continuous culturing experiments. Measurements not determined are indicated by “n.d.”. Concentrations below detection limit are indicated as “b.d.”

| **pressure (MPa)** | **time**  **(h)** | **dilution rate (h^-1^)** | **cells/mL**  ***10^7^** | | **NO_3_**  **(mM)** | | **NH_4_ (μM)** | **H_2_ (mM)** | **CO_2_ (mM)** | **fmol H_2_ / cell/hr** | | **fmol CO_2_ / cell/hr** | **δ^15^N-NH_4_ (‰)** | **δ^15^N-NO_3_ (‰)** |
| --- | --- | --- | --- | --- | --- | --- | --- | --- | --- | --- | --- | --- | --- | --- |
|  |  |  | **H_2_/CO_2_ (80:20) at 0.21 MPa in headspace of the medium bottle** | | | | | | | | | |  |  |
| 0.1 | 0.3 | 0.20 | 4.6 ± 1.13 | | 14.88 | | 160 | n.d. | n.d. | n.d. | | n.d. | n.d. | n.d. |
| 0.4 | 5.9 | 0.03 | 2.00 ± 0.66 | | 14.88 | | 301 | n.d. | n.d. | n.d. | | n.d. | n.d. | n.d. |
| 0.4 | 9.9 | 0.06 | 1.50 ± 0.45 | | n.d. | | n.d. | n.d. | n.d. | n.d. | | n.d. | n.d. | n.d. |
| 0.4 | 19.4 | 0.04 | 1.88 ± 1.51 | | 15.15 | | 326 | n.d. | n.d. | n.d. | | n.d. | n.d. | 24.3 ± 0.4 |
|  | 21.4 | setting the next dilution rate | | | | | | | | | | | | |
| 0.4 | 26.1 | 0.11 | 3.52 ± 0.97 | | 15.66 | | 237 | n.d. | n.d. | n.d. | | n.d. | n.d. | n.d. |
|  | 27.1 | setting the next dilution rate | | | | | | | | | | | | |
| 0.4 | 43.8 | 0.01 | 16.8 ± 2.48 | | 15.56 | | 99 | n.d. | n.d. | n.d. | | n.d. | n.d. | n.d. |
| 0.4 | 53.3 | 0.01 | 14.5 | | 15.02 | | 215 | n.d. | n.d. | n.d. | | n.d. | n.d. | n.d. |
|  | 53.5 | setting the next dilution rate | | | | | | | | | | | | |
| 0.4 | 70.5 | 0.06 | 2.75 | | 15.50 | | 159 | n.d. | n.d. | n.d. | | n.d. | n.d. | n.d. |
| 0.4 | 78.0 | 0.06 | 3.20 | | 15.43 | | 219 | n.d. | n.d. | n.d. | | n.d. | n.d. | n.d. |
|  | 79.0 | setting the next dilution rate | | | | | | | | | | | | |
| 0.4 | 94.5 | 0.11 | 9.50 | | 14.92 | | 398 | n.d. | n.d. | n.d. | | n.d. | n.d. | n.d. |
| 0.4 | 101.5 | 0.11 | 5.50 | | 14.69 | | 335 | n.d. | n.d. | n.d. | | n.d. | n.d. | n.d. |
| 0.4 | 117.8 | 0.11 |  | | 15.15 | | 271 | n.d. | n.d. | n.d. | | n.d. | -4.2 ± 2.7 | 27.6 ± 2.9 |
| 0.4 | 123.8 | 0.12 | 30.6 ± 4.01 | | 14.77 | | 295 | <0.01 | 6.50 | 0.5 ± 0.1 | | 2.7 ± 0.4 | -5.4 ± 3.9 | n.d. |
| 10 | 126.0 | setting the next dilution rate | | | | | | | | | | | | |
| 10 | 141.8 | 0.06 | 14.2 ± 2.44 | | 14.54 | | 518 | 0.01 | 6.38 | 0.5 ± 0.1 | | 3.4 ± 0.6 | -5.1 ± 3.6 | 26.0 ± 1.3 |
|  | 142.0 | setting the next dilution rate | | | | | | | | | | | | |
| 10 | 149.6 | 0.11 | 10.1 ± 2.06 | | 14.39 | | 583 | b.d. | 5.14 | n.d. | | 11.9 ± 2.4 | n.d. | n.d. |
|  | 149.8 | setting the next dilution rate | | | | | | | | | | | | |
| 10 | 163.9 | 0.06 | 12.9 ± 2.71 | | 14.37 | | 333 | n.d. | n.d. | n.d. | | n.d. | n.d. | n.d. |
| 10 | 165.1 | 0.09 | 12.9 ± 2.66 | | n.d. | | n.d. | n.d. | n.d. | n.d. | | n.d. | n.d. | n.d. |
| 20 | 166.1 | setting the next dilution rate | | | | | | | | | | | | |
| 20 | 171.7 | 0.11 | 9.34 ± 2.58 | | 14.57 | | 567 | n.d. | n.d. | n.d. | | n.d. | -3.4 ± 1.9 | 25.2 ± 0.5 |
| 20 | 177.6 | 0.12 | 10.7 ± 3.15 | | 14.46 | | n.d. | n.d. | n.d. | n.d. | | n.d. | n.d. | n.d. |
| 20 | 187.6 | 0.11 | 4.75 ± 1.43 | | 14.74 | | 291 | n.d. | n.d. | n.d. | | n.d. | n.d. | n.d. |
|  | 188.6 | setting the next dilution rate | | | | | | | | | | | | |
| 20 | 193.6 | 0.14 | 4.80 ± 1.88 | | 14.59 | | 288 | n.d. | n.d. | n.d. | | n.d. | -3.4 ± 2.0 | 26.0 ± 1.3 |
| 20 | 201.6 | 0.14 | 16.4 ± 1.85 | | 14.39 | | 335 | n.d. | n.d. | n.d. | | n.d. | n.d. | n.d. |
| 20 | 210.6 | 0.14 | 5.45 ± 1.86 | | 14.34 | | n.d. | n.d. | n.d. | n.d. | | n.d. | n.d. | n.d. |
|  |  |  | **H_2_/CO_2_ (80:20) at 0.31 MPa in headspace of the medium bottle** | | | | | | | | | |  |  |
| 20 | 218.0 | 0.14 | 13.5 ± 2.66 | 15.03 | | 466 | | 0.01 | 6.25 | | 2.0 ± 0.4 | 17 ± 3.3 | -1.4 ± 0.1 | 24.5 ± 0.2 |
| 20 | 224.2 | 0.14 | 25.4 ± 7.21 | 14.88 | | 432 | | b.d. | 7.85 | | n.d. | 8.2 ± 2.3 | -1.9 ± 0.4 | 27.3 ± 2.6 |
| 20 | 235.8 | 0.15 | 8.68 ± 2.51 | 15.41 | | 352 | | n.d. | n.d. | | n.d. | n.d. | -1.6 ± 0.2 | 25.5 ± 0.8 |
| 20 | 241.1 | 0.14 | 4.47 ± 1.14 | 16.12 | | 367 | | b.d. | 8.81 | | n.d. | 44 ± 11 | n.d. | n.d. |
|  | 242.1 | setting the next dilution rate | | | | | | | | | | | | |
| 20 | 244.1 | 0.28 | 4.33 ± 1.59 | 17.28 | | 435 | | 0.08 | 6.73 | | 12 ± 4.5 | 102 ± 38 | -1.5 ± 0.0 | 26.9 ± 2.2 |
| 20 | 248.2 | 0.28 | 5.88 ± 1.38 | 17.09 | | 425 | | 0.03 | 9.43 | | 9.2 ± 2.1 | 62 ± 15 | n.d. | n.d. |
| 20 | 257.3 | 0.27 | 12.3 ± 4.53 | 16.87 | | 549 | | <0.01 | 9.93 | | 4.3 ± 1.6 | 28 ± 10 | -1.9 ± 0.4 | 24.7 ± 0.0 |
| 20 | 260.8 | 0.28 | 5.40 ± 0.99 | 17.43 | | 449 | | n.d. | n.d. | | n.d. | n.d. | n.d. | n.d. |
| 20 | 264.0 | 0.28 | 15.8 ± 1.96 | 16.88 | | 663 | | n.d. | n.d. | | n.d. | n.d. | n.d. | n.d. |
| 20 | 265.4 | 0.33 | 38.8 ± 4.39 | 16.73 | | 679 | | n.d. | n.d. | | n.d. | n.d. | n.d. | n.d. |
| 20 | 266.3 | 0.34 | 38.5 ± 6.45 | 16.58 | | 695 | | n.d. | n.d. | | n.d. | n.d. | n.d. | n.d. |
| 20 | 268.1 | 0.30 | 4.63 ± 1.50 | 16.43 | | 685 | | n.d. | n.d. | | n.d. | n.d. | n.d. | n.d. |
| 20 | 271.8 | 0.28 | 115 ± 15.3 | 17.20 | | 704 | | n.d. | n.d. | | n.d. | n.d. | n.d. | n.d. |
| 20 | 285.8 | 0.27 | 38.0 ± 4.24 | 17.50 | | 633 | | n.d. | n.d. | | n.d. | n.d. | n.d. | n.d. |
|  | 286.0 | setting the next dilution rate | | | | | | | | | | | | |
| 20 | 310.5 | 0.06 | 4.63 ± 0.97 | 17.21 | | 596 | | n.d. | n.d. | | n.d. | n.d. | n.d. | n.d. |
| 20 | 506.0 | 0.05 | 7.95 | 16.55 | | 671 | | n.d. | n.d. | | n.d. | n.d. | n.d. | n.d. |
| 20 | 596.7 | 0.26 | 10.5 ± 2.13 | 18.02 | | 294 | | n.d. | n.d. | | n.d. | n.d. | n.d. | n.d. |
| 20 | 598.8 | 0.29 | 29.1 ± 2.85 | 17.02 | | 549 | | n.d. | n.d. | | n.d. | n.d. | n.d. | n.d. |
| 20 | 600.8 | 0.30 | 36.9 ± 5.63 | 17.18 | | 419 | | n.d. | n.d. | | n.d. | n.d. | n.d. | n.d. |
| 20 | 602.8 | 0.31 | 24.3 ± 3.80 | 15.92 | | 471 | | n.d. | n.d. | | n.d. | n.d. | n.d. | n.d. |
| 20 | 605.0 | 0.29 | 29.6 ± 2.66 | 16.82 | | 390 | | n.d. | n.d. | | n.d. | n.d. | n.d. | n.d. |
| 20 | 624.2 | 0.26 | 24.7 ± 4.59 | 16.95 | | 687 | | n.d. | n.d. | | n.d. | n.d. | n.d. | n.d. |
| 20 | 626.2 | 0.30 | 35.8 ± 7.07 | 17.39 | | 671 | | n.d. | n.d. | | n.d. | n.d. | -2.4 ± 0.9 | 29.7 ± 5.0 |
| 20 | 628.2 | 0.31 | 17.2 ± 1.29 | 17.52 | | 753 | | n.d. | n.d. | | n.d. | n.d. | n.d. | n.d. |
| 20 | 644.0 | 0.27 | 21.1 ± 2.48 | 17.79 | | 506 | | n.d. | n.d. | | n.d. | n.d. | -0.8 ± 0.6 | 29.7 ± 5.0 |
|  | 645.0 | setting the next dilution rate | | | | | | | | | | | | |
| 20 | 646.5 | 0.42 | 28.5 ± 2.71 | 16.95 | | 434 | | <0.01 | 9.39 | | 2.8 ± 0.3 | 18 ± 1.7 | -1.7 ± 0.2 | 25.9 ± 1.2 |
| 20 | 648.5 | 0.43 | 23.0 ± 1.80 | 17.22 | | n.d. | | b.d. | 8.85 | | n.d. | 24 ± 1.9 | n.d. | n.d. |
| 20 | 650.3 | 0.44 | 22.2 ± 1.59 | 16.93 | | 395 | | 0.85 | 8.94 | | 2.1 ± 0.2 | 26 ± 1.8 | n.d. | n.d. |
| 20 | 652.3 | 0.43 | 8.05 ± 1.28 | n.d. | | 359 | | n.d. | n.d. | | n.d. | n.d. | -1.6 ± 0.1 | 26.9 ± 2.2 |
| 20 | 668.3 | 0.40 | 27.1 ± 3.81 | 17.21 | | 372 | | 0.16 | 6.73 | | 2.6 ± 0.4 | 22 ± 3.1 | -2.1 ± 0.6 | 27.9 ± 3.2 |
| 20 | 670.3 | 0.43 | 61.2 ± 6.77 | 17.06 | | 331 | | 0.15 | 7.88 | | 1.2 ± 0.1 | 9.6 ± 1.1 | n.d. | n.d. |
| 20 | 672.3 | 0.44 | 45.5 ± 11.6 | 17.19 | | 291 | | 0.19 | 7.73 | | 1.7 ± 0.4 | 14 ± 3.4 | n.d. | n.d. |
| 20 | 674.3 | 0.43 | 20.9 ± 3.35 | 17.20 | | 243 | | 1.60 | 8.67 | | 0.6 ± 0.1 | 27 ± 4.3 | n.d. | n.d. |
| 20 | 692.2 | 0.40 | 32.5 ± 2.60 | 17.57 | | 258 | | 0.20 | 8.61 | | 2.1 ± 0.2 | 16 ± 1.3 | n.d. | n.d. |
|  | 692.4 | setting the next dilution rate | | | | | | | | | | | | |
| 20 | 694.2 | 0.57 | 23.5 ± 4.24 | 17.64 | | 222 | | 0.32 | 7.55 | | 3.8 ± 0.7 | 34 ± 6.2 | n.d. | n.d. |
| 20 | 696.2 | 0.52 | 22.7 ± 3.07 | 17.98 | | 204 | | 0.36 | 7.51 | | 3.5 ± 0.5 | 33 ± 4.4 | -2.6 ± 1.1 | 24.2 ± 0.5 |
| 20 | 698.2 | 0.59 | 31.5 ± 4.18 | 17.07 | | 397 | | 0.34 | 6.86 | | 2.9 ± 0.4 | 28 ± 3.7 | -2.0 ± 0.5 | 26.4 ± 1.7 |
|  | 699.0 | setting the next dilution rate | | | | | | | | | | | | |
| 20 | 812.2 | 0.05 | 38.6 ± 5.72 | 14.72 | | 791 | | n.d. | n.d. | | n.d. | n.d. | n.d. | n.d. |
|  | 813.0 | setting the next dilution rate | | | | | | | | | | | | |
| 20 | 814.3 | 0.69 | 7.50 ± 1.45 | 14.01 | | 610 | | 1.10 | 6.65 | | 7.4 ± 1.4 | 139 ± 27 | n.d. | n.d. |
| 20 | 816.3 | 0.69 | 6.60 ± 1.41 | 14.01 | | 542 | | 1.82 | 7.12 | | 0.8 ± 0.2 | 153 ± 33 | n.d. | n.d. |
|  | 817.0 | setting the next dilution rate | | | | | | | | | | | | |
| 20 | 818.3 | 0.83 | 32.2 ± 3.53 | 13.34 | | 688 | | 0.80 | 6.66 | | 2.8 ± 0.3 | 39 ± 4.2 | -2.8 ± 1.3 | n.d. |
| 20 | 820.3 | 0.83 | 65.7 ± 7.05 | 13.56 | | 823 | | <0.01 | 5.05 | | 2.4 ± 0.3 | 21 ± 2.2 | n.d. | n.d. |
|  | 820.5 | setting the next dilution rate | | | | | | | | | | | | |
| 20 | 836.1 | 0.27 | 4.80 ± 1.22 | 13.66 | | 814 | | n.d. | n.d. | | n.d. | n.d. | n.d. | n.d. |
|  | 836.7 | setting the next dilution rate | | | | | | | | | | | | |
| 20 | 838.3 | 1.09 | 11.0 ± 1.53 | 13.80 | | 608 | | 0.95 | 6.26 | | 9.3 ± 1.3 | 152 ± 21 | n.d. | n.d. |
| 20 | 840.3 | 1.07 | 3.78 ± 0.80 | 14.04 | | 529 | | 0.86 | 4.96 | | 30 ± 6.3 | 476 ± 101 | n.d. | n.d. |
| 20 | 842.3 | 1.09 | 5.33 ± 1.14 | 14.36 | | 549 | | 1.04 | 8.94 | | 17 ± 3.7 | 260 ± 56 | -2.1 ± 0.6 | 28.5 ± 3.8 |
|  | 938.3 | setting the dilution rate at 0.005 h^-1^ | | | | | | | | | | | | |
|  | 951.7 | setting the next dilution rate | | | | | | | | | | | | |
| 20 | 957.7 | 0.52 | 8.43 ± 0.86 | 13.23 | | 522 | | n.d. | n.d. | | n.d. | n.d. | n.d. | n.d. |
|  |  | setting the next dilution rate | | | | | | | | | | | | |
| 20 | 960.0 | 1.30 | 8.40 ± 1.40 | n.d. | | 673 | | 0.02 | 0.26 | | 29 ± 4.9 | 333 ± 55 | n.d. | n.d. |
| 20 | 962.0 | 1.30 | 31.0 ± 24.4 | 13.14 | | 482 | | 0.01 | 5.50 | | 7.9 ± 6.3 | 68 ± 54 | n.d. | n.d. |
| 20 | 966.2 | 1.30 | 12.4 ± 3.53 | 12.08 | | 416 | | <0.01 | 4.83 | | 20 ± 5.7 | 177 ± 50 | n.d. | n.d. |
|  | 966.4 | setting the dilution rate at 0.005 h^-1^ | | | | | | | | | | | | |
|  | 981.2 | setting the next dilution rate | | | | | | | | | | | | |
| 20 | 984.3 | 0.52 | 11.0 ± 1.30 | 12.39 | | 542 | | 0.69 | 4.95 | | 5.8 ± 0.7 | 80 ± 10 | n.d. | n.d. |
|  |  | setting the next dilution rate | | | | | | | | | | | | |
| 20 | 986.3 | 1.57 | 15.5 ± 1.96 | 12.14 | | 677 | | 0.10 | 5.97 | | 18 ± 2.3 | 159 ± 20 | n.d. | n.d. |
| 20 | 988.3 | 1.57 | 26.6 ± 3.72 | 13.33 | | 575 | | n.d. | 4.61 | | n.d. | 101 ± 14 | n.d. | n.d. |
| 20 | 990.3 | 1.57 | 6.05 ± 1.46 | 12.05 | | 615 | | <0.01 | 6.96 | | 49 ± 12 | 381 ± 92 | n.d. | n.d. |
|  | 991.3 | setting the dilution rate at 0.005 h^-1^ | | | | | | | | | | | | |
|  | 1006.3 | setting the next dilution rate | | | | | | | | | | | | |
| 20 | 1009.8 | 0.52 | 7.55 ± 1.44 | 12.99 | | 498 | | 0.12 | 5.9 | | 12 ± 2.3 | 110 ± 21 | n.d. | n.d. |
|  | 1010.3 | setting the next dilution rate | | | | | | | | | | | | |
| 20 | 1012.2 | 2.11 | 118 ± 13.7 | 12.34 | | 716 | | <0.01 | 5.9 | | 3.4 ± 0.4 | 28 ± 3.2 | n.d. | n.d. |
| 20 | 1014.2 | 2.12 | 26.3 ± 2.82 | 13.42 | | 511 | | <0.01 | 5.6 | | 15.3 ± 1.6 | 129 ± 14 | -2.6 ± 1.1 | 26.6 ± 1.9 |
| 20 | 1015.6 | 2.13 | 8.63 ± 1.96 | 13.48 | | 452 | | <0.01 | 6.2 | | 47 ± 11 | 383 ± 87 | n.d. | n.d. |
|  | 1016.6 | setting the dilution rate at 0.005 h^-1^ | | | | | | | | | | | | |
|  | 1028.0 | setting the next dilution rate | | | | | | | | | | | | |
| 20 | 1029.6 | 0.53 | 7.10 ± 1.98 | 13.85 | | 334 | | n.d. | n.d. | | n.d. | n.d. | n.d. | n.d. |
|  | 1030.0 | setting the next dilution rate | | | | | | | | | | | | |
| 20 | 1031.7 | 2.61 | 6.70 ± 2.40 | 13.72 | | 172 | | 0.99 | 6.5 | | 35 ± 13 | 589 ± 211 | n.d. | n.d. |
| 20 | 1033.7 | 2.61 | 9.45 ± 2.47 | 15.36 | | 202 | | 0.65 | 5.8 | | 35 ± 9.0 | 437 ± 114 | n.d. | n.d. |
| 20 | 1035.5 | 2.61 | 5.56 ± 0.93 | 15.99 | | 380 | | 0.36 | 4.5 | | 72 ± 12 | 809 ± 135 | -3.3 ± 1.8 | n.d. |
| 20 | Dilution rate set at 0.78 h^-1^ to allow biomass retrieval for proteomics | | | | | | | | | | | | | |
| 0.5 | 1460.0 | setting the next dilution rate | | | | | | | | | | | | |
| 0.5 | 1461.5 | 0.05 | 0.90 ± 0.27 | 10.23 | | 780 | | b.d. | 7.3 | | n.d. | 83 ± 25 | n.d. | n.d. |
|  | 1463.5 | setting the next dilution rate | | | | | | | | | | | | |
| 0.5 | 1465.8 | 0.22 | 18.9 ± 3.47 | 10.56 | | 286 | | b.d. | 5.8 | | n.d. | 19 ± 3.4 | n.d. | n.d. |
| 0.5 | 1469.8 | 0.23 | 30.9 ± 5.21 | 9.68 | | 651 | | 0.36 | 7.2 | | 1.1 ± 0.2 | 11 ± 1.8 | n.d. | n.d. |
| 0.5 | 1485.8 | 0.21 | 18.6 ± 4.04 | 10.90 | | 196 | | 0.22 | 5.6 | | 1.9 ± 0.4 | 19 ± 4.0 | n.d. | n.d. |
|  | 1486.0 | setting the next dilution rate | | | | | | | | | | | | |
| 0.5 | 1487.8 | 0.56 | 16.4 ± 1.47 | 10.86 | | 381 | | 0.34 | 4.5 | | 5.3 ± 0.5 | 59 ± 5.3 | n.d. | n.d. |
| 0.5 | 1489.8 | 0.56 | 11.8 ± 1.60 | 9.73 | | 698 | | 0.64 | 7.0 | | 6.0 ± 0.8 | 70 ± 9.5 | n.d. | n.d. |
| 0.5 | 1491.8 | 0.55 | 47.8 ± 10.3 | 10.76 | | 262 | | 0.58 | 6.6 | | 1.5 ± 0.3 | 17 ± 3.8 | n.d. | n.d. |
|  | 1492.3 | setting the next dilution rate | | | | | | | | | | | | |
| 0.5 | 1509.8 | 0.26 | 15.0 ± 2.43 | 10.63 | | 313 | | 0.27 | 5.7 | | 2.9 ± 0.5 | 28 ± 4.6 | n.d. | n.d. |
|  | 1510.8 | setting the next dilution rate | | | | | | | | | | | | |
| 0.5 | 1512.2 | 0.80 | 20.7 ± 2.99 | 10.22 | | 388 | | 0.61 | 3.3 | | 5.0 ± 0.7 | 72 ± 10 | n.d. | n.d. |
| 0.5 | 1514.5 | 0.81 | 21.3 ± 3.48 | 9.64 | | 340 | | 0.81 | 4.4 | | 4.1 ± 0.7 | 66 ± 11 | n.d. | n.d. |
| 0.5 | 1516.1 | 0.82 | 74.0 ± 8.56 | 9.68 | | 280 | | 0.66 | 4.2 | | 1.4 ± 0.2 | 19 ± 2.2 | n.d. | n.d. |
|  | 1516.3 | setting the next dilution rate | | | | | | | | | | | | |
| 0.5 | 1533.4 | 0.27 | 4.73 ± 1.03 | 9.47 | | 314 | | 0.25 | 5.7 | | 9.4 ± 2.1 | 91 ± 20 | n.d. | n.d. |
| 0.5 | 1535.4 | 1.07 | 13.8 ± 1.77 | 9.59 | | 310 | | 0.83 | 5.4 | | 8.3 ± 1.1 | 127 ± 16 | n.d. | n.d. |
| 0.5 | 1537.4 | 1.10 | 43.0 ± 5.20 | 9.59 | | 295 | | 0.99 | 6.6 | | 2.3 ± 0.3 | 39 ± 4.7 | n.d. | n.d. |
| 0.5 | 1539.2 | 1.11 | 18.9 ± 4.25 | 9.78 | | 258 | | 0.67 | 2.8 | | 7.2 ± 1.6 | 111± 25 | n.d. | n.d. |
|  | 1540.0 | setting the next dilution rate | | | | | | | | | | | | |
| 0.5 | 1629.2 | 0.5 | 1.71 ± 0.45 | n.d. | | n.d. | | 0.02 | 5.7 | | 5.9 ± 1.5 | 50 ± 13 | n.d. | n.d. |
|  | 1629.3 | setting the next dilution rate | | | | | | | | | | | | |
| 0.5 | 1631.7 | 1.60 | 6.43 ± 0.72 | 9.38 | | 305 | | 1.46 | 4.6 | | 11.0 ± 1.2 | 424 ± 47 | n.d. | n.d. |
| 0.5 | 1633.3 | 1.60 | 1.43 ± 4.42 | 9.31 | | 234 | | 1.48 | 4.6 | |  |  | n.d. | n.d. |
